# Will there be new trends in the public’s attention to express services in the post-COVID-19 era?

**DOI:** 10.1371/journal.pone.0348096

**Published:** 2026-06-24

**Authors:** Xin Chen, Jie Gao, Qin Qin, Chuanlei Wang

**Affiliations:** 1 School of Business, Anhui University, Hefei, PR China; 2 Anhui Zhong-Ao Institute of Technology, Hefei, PR China; Max Planck Institute for Solid State Research, GERMANY

## Abstract

To explore the evolution trends and sentiment characteristics of public’s attention toward express services following the comprehensive adjustment of COVID-19 prevention policies in China, and to provide empirical evidence for service quality improvement and regulatory policy optimization, this paper uses Weibo data from December 10, 2022, to January 10, 2023 as research samples. This paper employs the Entropy Weight Method (EWM) to quantify post popularity and identify high-attention content, applies the Dynamic Topic Modeling (DTM) to uncover temporal patterns of public attention themes, and constructs a CNN-LSTM model for sentiment analysis to reveal cognitive differences between official certification bloggers and personal certification bloggers. The findings include: (1) Public sentiment toward express services was predominantly negative, and sentiment fluctuations were highly correlated with logistics pressure events such as the “Double 12” shopping festival, pandemic infection peaks, and the New Year’s Day holiday; (2) Six core topics were identified, with “goods safety” and “after-sales service” representing persistent traditional attention, while “courier rights” and “governments’ requirements for enterprises” emerged as two new trends in the post-pandemic era; (3) Official certification bloggers adopted a macro perspective focusing on enterprise service quality and logistics economy, whereas personal certification bloggers emphasized micro-level experiences regarding last-mile delivery conflicts and labor rights, with both groups converging on the issue of “courier rights.” In the post-COVID-19 era, public’s attention to express services has extended beyond efficiency and quality demands to encompass deeper dimensions such as labor rights protection and corporate social responsibility. Express enterprises should strengthen courier team building and optimize service processes, while governments need to improve industry standard-setting and regulatory incentive mechanisms to promote sustainable industry development.

## Introduction

With the rapid rise of e-commerce, China’s express service industry has experienced rapid development in recent years. According to the data from the State Post Bureau of China, the business volume of China’s express service industry has grown rapidly in the past 14 years, from 1.86 billion parcels in 2009 to 132.07 billion parcels in 2023, an increase of 71 times, ranking first in the world for ten consecutive years, and leading global express changes. At the same time, in 2023, the satisfaction and punctuality rate of China’s express services greatly improved. The 72-hour delivery rate of express services reached 80.97%, and the public satisfaction score and the 72-hour delivery rate of express services both reached their best historical levels. However, some problems affect the sustainable development of China’s express service industry. Based on complaints of postal industry users in the fourth quarter of 2023 released by the State Post Bureau, there were 68,956 complaints about express services, of which 11,081 were valid complaints, accounting for 16.07%. The main issues involved in valid complaints are the loss or shortage of parcels, delivery service, and damage to parcels, accounting for 36.39%, 27.49%, and 18.64% of total valid complaints, respectively. Therefore, how to improve the level of express services, strengthen the service standards, and enhance the publics satisfaction with the services of express enterprises remain the focus of research in the express service industry.

From an international perspective, cross-border control measures triggered by the pandemic have directly impacted the core operational chains of international express delivery. A Universal Postal Union report shows that global international mail (including small parcels), postal parcels, and EMS volumes under 2 kg continued their fluctuating downward trend in 2022, with European mail imports declining by 58%. Specific manifestations of supply chain disruptions include: a persistent shortage of air cargo capacity, reduced capacity on intercontinental routes such as Asia-Europe and Asia-Africa, and frequent port and sorting center shutdowns due to infection. The EU’s newly implemented Electronic Advance Declaration Data (EAD) regulations and the operation of the Import Control System (ICS2) have further exacerbated customs clearance delays. This decline in service stability has directly driven up complaint rates, echoing issues such as lost and delayed packages in my country.

China has a large online consumer market [1]. In 2023, the per capita express volume in China exceeded 90 packages, indicating strong public demand for express. The public’s demand for express deliveries determines the development direction of the express service industry. Therefore, identifying new trends in the public’s attitude to express services and seizing the initiative will provide insights into the development of China’s express service industry.

In December 2019, the sudden outbreak of COVID-19 had an enormous impact on China. The outbreak of the epidemic in Wuhan marked the moment when domestic COVID-19 cases were no longer contained. To curb the spread of the virus, Wuhan imposed a lockdown. From 1 January 2020 to 30 June 2021, COVID-19 caused heavy losses worldwide [2]. From February to April 2022, the number of people infected across the country increased rapidly as the epidemic surged. On December 9, 2022, news about the official end of COVID-19 was announced, marking 3 years of pandemic conditions.

When COVID-19 was widespread, it caused a major setback to the development of the logistics and transportation industry in particular [3,4]. Moreover, many consumers started shopping online in response to measures such as city lockdown [5]. Owing to being confined at home, people were able to obtain the necessary goods only through services [6]. Express services, as a vital part of the express service industry, have become a hot topic of public discussion. As of June 10, 2020, China’s postal and express enterprises had delivered 470,300 tons of epidemic prevention and control materials and 388 million packages [7]. Therefore, understanding the public’s attention and attitudes toward express services is crucial for promoting the sustainable development of the express service industry. Domestic and international scholars have analyzed the public’s attitudes toward to express services before and during the pandemic. Zhang et al. [8] captured the online comments of 8 express enterprises in Baidu Koubei from 2015 to 2017 and studied customer satisfaction with express services through an LDA topic model and sentiment analysis to identify new methods of express service innovation. Li [9] collected customer review data from 10 express enterprises in Baidu Koubei in 2019 and analyzed the main factors affecting customer satisfaction with express services. Zheng et al. [10] analyzed questionnaires collected from July 2021 to August 2021 to determine the factors affecting customer satisfaction and loyalty in terms of express service quality. Ding [11] used a semantic network model and LDA topic model to identify problems in express services. The above studies are based on public perspectives to determine the public’s attitude to the field of express services. With the full lifting of COVID-19 restrictions, due to changes in public consumption habits, behaviors and attitudes, the public’s attitude to express services in China has also changed. As an important bridge connecting online and offline services, all aspects of express services have received widespread attention. Few scholars have researched and analyzed the public’s attitudes toward express services after the full lifting of COVID-19 restrictions. Therefore, studying the new trends and attention of the public to express services after the full lifting of COVID-19 restrictions is highly important for improving the service level of the express services industry and meeting public demands.

In recent years, more members of the public have used social media to share their opinions (perceptions, ideas, and comments) at any time [12]. Logistics companies can accurately determine consumers’ attitudes toward services by analyzing comments on social media. It is possible to quickly obtain public comments on express services through social media [13]. Social media channels are important for the reputations of organizations, products and services [14]. This paper aims to determine the public’s attention to express services post-COVID-19.Therefore, the following research is proposed.

(1) By analyzing social media data, identify new trends in attention and determine how public focus on express services has shifted after the lifting of COVID-19 restrictions, and compare these focus areas with those prevalent before and during the pandemic.
(2) Analyze the public’s sentiment tendencies (positive or negative) towards different period after the lifting of COVID-19 restrictions to reveal their levels of satisfaction and key areas of dissatisfaction. By this way, it can gain insights into public sentiment attitudes.
(3) Based on the research findings, offer specific management suggestions for express service enterprises to enhance their service levels and sustainable development capabilities, while also providing a reference for the government to formulate and improve policies related to the service industry. Most researchers analyze and optimize express services from the viewpoint of the express service industry, but there is a lack of research on the views of public’s attention. This paper starts from a public perspective, and uses microblog to obtain the public’s attention about express services to better understand the public’s demands for express services.

## Related works

### The overall theoretical framework

**Fig 1:**
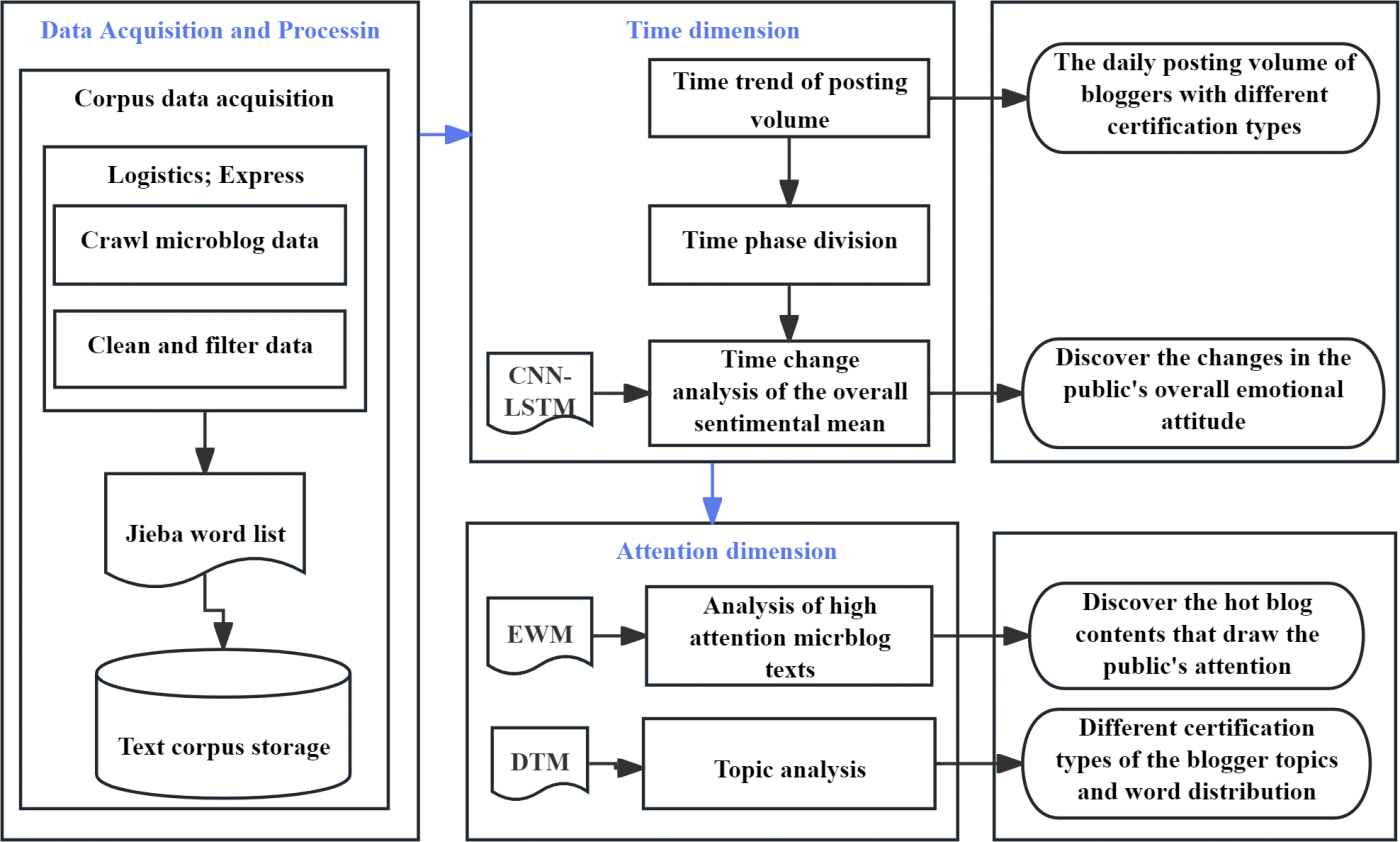
The overall theoretical framework.

This paper aims to explore the public’s attention to express services from the perspective of different types of bloggers. Fig 1 shows the overall theoretical framework.

The overall theoretical framework is a multi-dimensional, multi-method research framework based on social media data analysis, aimed at exploring new trends in public attention towards express services in the post-COVID-19 era. This framework integrates technologies such as text mining, topic modeling, and sentiment analysis to systematically analyze changes in public attention from both time and attention dimensions. Specifically, the framework consists of the following core components:

#### Data layer.

Based on data from the microblog social media platform, public expressions regarding express services are collected through keyword crawling and text preprocessing (such as word segmentation and stop-word removal).

#### Analysis layer.

Time Dimension Analysis: Peaks in posting volume and fluctuations in sentiment values are linked to real-world events (such as the “Double 12” shopping festival and peaks in pandemic infections). This connection is analyzed through the temporal dimension within the theoretical framework to explain the reasons behind changes in public attention intensity.

Attention Dimension Analysis: (1) Attention Measurement: Uses the Entropy Weight Method (EWM) to objectively quantify the popularity of microblog texts (based on weights assigned to retweets, comments, and likes), identifying high-attention content. (2) Sentiment Analysis: Employs a CNN-LSTM model to perform sentiment classification on microblog texts, assessing public sentiment attitudes towards express services. (3) Topic Mining: Applies the DTM to extract latent topics from microblog texts, revealing the core issues of public’s attention.The topics extracted using the DTM were compared with literature research from different periods of the COVID-19 pandemic, identifying new trends in public’s attention during the post-pandemic era. This longitudinal comparison of topic evolution reveals shifts in public focus after the pandemic.

The above methods can help determine public’s attention to express services post-COVID-19, identify potential problems in express services, and enable an analysis of these problems, providing input for companies to improve their express service quality.

### Express services

Express services originated from letter delivery services [15]. On January 1, 2008, the “Express Service” implemented by the State Post Bureau of China defined express services as the rapid receipt, transportation, delivery of individually packaged, addressed express or other items that do not need to be stored, delivered to the recipient or a designated place within the promised time limit, and received by delivery service [16]. Through express delivery, goods can be delivered to customers in different places [17,18]. With the emergence of new technologies, the express industry is developing rapidly, and the needs of customers for express services have gradually diversified [8]. To improve sustainable development, the level of express services has a significant impact on the satisfaction of e-commerce customers [19]. Zhuo and Miao [20] concluded that due to the particularity of China’s express delivery industry, “security” and “timeliness” are important additional dimensions of service quality. These qualities include relatively high delivery prices, unreasonable signing process, large timeliness of delivery, delayed delivery, damage and loss of goods, signing before inspection etc in express services, which led to complaints from customers [21,22]. From the public perspective, this paper analyses the situation of public’s attention to express services post-COVID-19, and determines whether there have been new changes in the public’s attention to express services. It is necessary to improve the concrete aspects of express services and cater to the demands of the public.

### Social media data

The booming development of big data and data-mining technologies has enabled the exploitation of social media as a source of public information [23], e.g., the use of text mining technology to automatically and quickly mine trending keywords in public opinion and emotional tendencies from massive microblog text data. Social media data are unstructured vast in quantity, and generate a large quantity of quantifiable valuable data for researchers and managers [24]. In the era of converged media, an increasing number of people express their views and opinions on social media platforms (such as blogs, forums, social networks, etc.) by posting blogs and comments [25]. The Sina Microblog is one of the most popular social media platforms in China [26]. In 2022, the number of monthly active users with microblogs reached 586 million, and the number of daily active users reached 252 million. With their enormous user bases and high activity, microblogs have become an important social media platform for information exchange in China about the COVID-19 epidemic. Sina is a microblogging service similar to Twitter and Facebook [27]. Users utilize microblog functions (e.g., replies, the @ function) to interact with each other, forming a rich corpus of user behavior data [28]. With in-depth analysis of microblog text data, insights into public’s attention to express services can be gained.

## Methodology

### Data acquisition and preprocessing

#### Data acquisition.

Express services are a hot content of public’s attention.After the full lifting of COVID-19 restrictions,improving the level of express services through the study of public’s attention and changes is highly important. To accurately capture the sudden impact of policy shift on the express delivery industry and obtain immediate public feedback on express services, this paper uses “express” and “logistics” as keywords to crawl data from microblog within one month from December 10, 2022, to January 10, 2023. The complete code is publicly available at https://github.com/yyinchen/COVID-19-Sentiment-analysis.git. All data collected for this paper strictly adheres to the Terms and Conditions of web-crawling from Weibo. A total of 155,951 relevant popular microblog texts were obtained. During this period, the express delivery industry faced multiple shocks: initial infection of couriers leading to staff shortages, the combined effects of the “Double 12” shopping festival and the fluctuating demand during the New Year’s Day holiday. Public feedback on express delivery services during this period is both immediate and representative, effectively reflecting the actual impact of policy adjustments on the industry.

#### Data preprocessing.

Text preprocessing mainly includes building a user dictionary, removing stopwords, word segmentation, and extracting keywords using the Jieba.The Harbin Institute of Technology Chinese Dictionary and Baidu Dictionary (including common Chinese vocabulary and part-of-speech tagging rules) were incorporated to cover word segmentation needs for daily language;Manually organize core terms in the express field, including business process terms (such as “sorting center,” “transfer station,” and “door-to-door pickup”), company names (such as “JD,” “SF Express,” and “Cainiao Station”), and service issues (such as “violent sorting,” “package backlog,” and “logistics stagnation”);This paper also supplemented the vocabulary with high-frequency post-pandemic terms, such as “ibuprofen,” “antigen testing,” “disinfection,” and “epidemic prevention supplies,” to adapt to the context of the research period (December 2022-January 2023).

Jieba is used to segment the posts and translate them into a list of meaningful words, including different lexical classes, such noun, verb, adv and adj [29]. In the process of data processing meaningless mood particles,are removed and repetitive texts and punctuation marks are deleted.Use common stopwords, including modal particles without actual meaning (such as “啊”, “呢”, “啦”), auxiliary words (such as “的”, “地”, “得”), punctuation marks (such as “!”, “?”, “...”), numbers and letters (such as “123”, “abc”); based on the characteristics of the microblog platform, supplement with forwarding symbols (such as “@XXX”, “forward”, “//”), topic tags (such as the “#” symbol and non-core topic words in “#北京抗疫#”), and emoticons. After cleaning and processing, a total of 146,584 texts were obtained. Examples of the resulting data samples are shown in Table 1.

**Table 1:** Example of microblog texts after text preprocessing.

Microblog certification	Original blog	Microblog text after data preprocessing
Official	[Thanks for your hard work!] Recently, reinforcement couriers from all over the country have arrived in Beijing one after another to start work. “Everyone, don’t worry, we are here!” Thank you, and please protect yourself! L People’s Daily’s microblog video	Arriving in Beijing/Express delivery/Reinforcement/Successive/microblog/Everywhere/Thank you/People’s Daily/Video/Hard work/Devotion/Recently/Protection/Don’t/Everyone/Work/Myself/We/
Personal	Beijing turns into Yangcheng: Manpower shortage in all walks of life! Supermarkets, pharmacies, and express delivery all have unique tricks #Beijing epidemic##Are you sunny today# 2 Beijing L Li Guangdou’s microblog video	Beijing/Li Guangdou/Niyang/microblog/Yangcheng/Express/All walks oflife/ Pharmacy/Surprise/Manpower/Shortage/Epidemic/Video/Supermarket/Today/

### Time dimension analysis

This paper’s time-series analysis consists of two parts: trends in posting volume and trends in mean sentiment.The goal is to capture the dynamic patterns of public interest in express services after the full lifting of COVID-19 restrictions from the dual dimensions of “attention intensity” and “sentiment orientation” providing support for the research conclusions regarding public attention trends.The post volume trend quantifies the public’s “attention intensity” for express services through fluctuations in posting volume and correlates it with external events (epidemics, holidays, and industry conditions) to find key points of public attention.The mean sentiment trend captures the evolution of public “sentiment orientation” toward express services through fluctuations in mean sentiment and, combining posting volume trends with external events, identifies the underlying causes of these sentiments.

### Attention dimension analysis

#### EWM.

Lu and Liu [30] proposed a denoising model based on the entropy weight method (EWM) to filter the noise and select the most relevant sentences. The EWM sorts the microblog, and obtains the hot texts that receive the most attention. The number of retweets, likes and comments on microblog can reflect the popularity of the microblog and the degree of public’s attention it receives. However, microblog forwarding, likes, and comments, differ in order of magnitude, as does the associated difficulty for users in executing these actions. Therefore, these three indicators need to be combined to identify popular microblogs and hot texts, more rigorously and reasonably. Because the factor judgment method and AHP are relatively subjective, this paper introduces a more objective EWM and uses the amount of information provided by the entropy value of each index to determine the weight of the index.

#### DTM.

**Fig 2:**
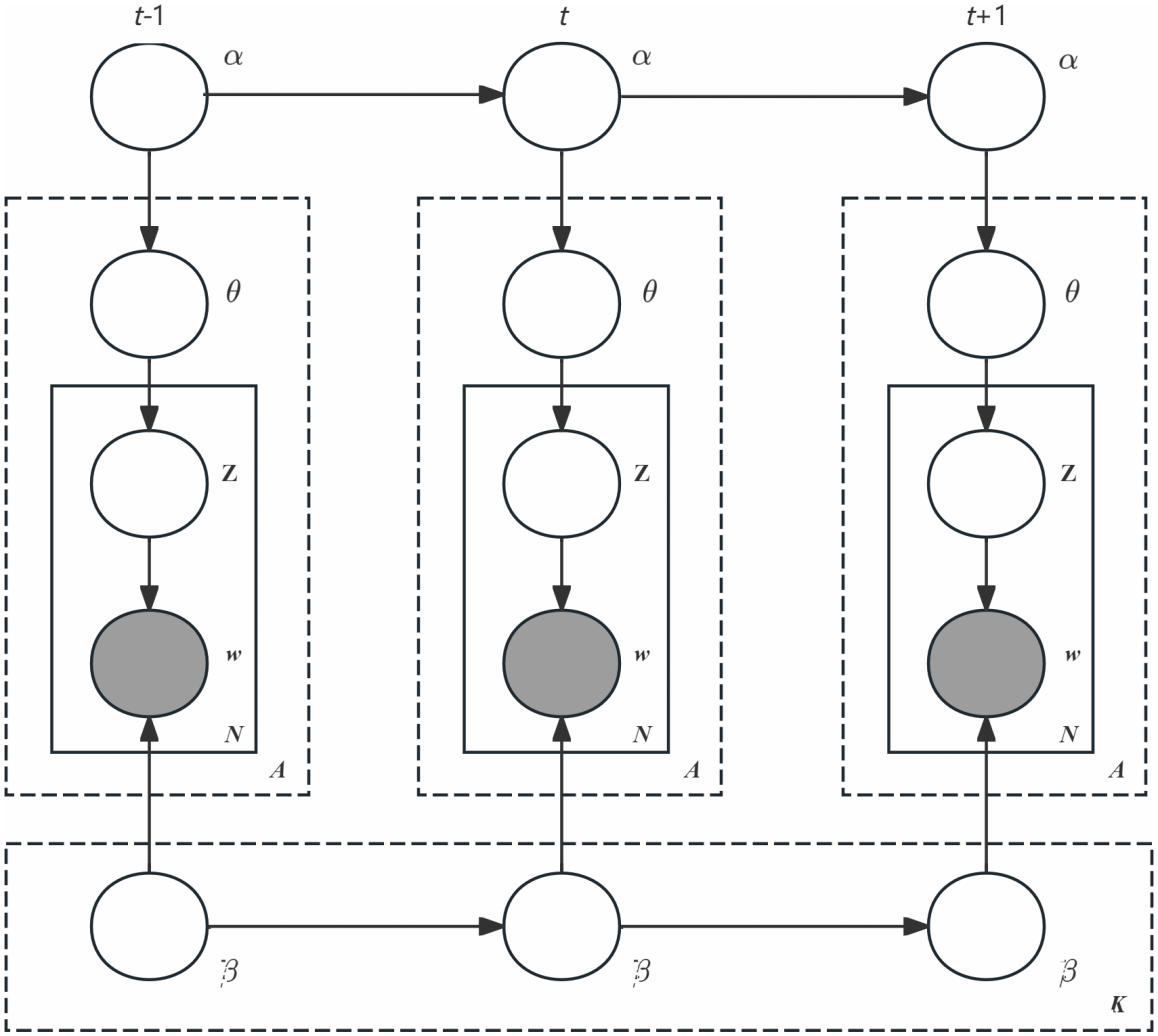
Dynamic topic modeling.

This paper adopts the Dynamic Topic Modeling (DTM) proposed by Blei et al. [31] for modeling. It is an unsupervised machine learning topic generation model improved on the basis of the Latent Dirichlet Allocation (LDA) model [32]. Compared to the LDA model, the DTM assumes that topics evolve over time, thus simulating the evolution of word distribution and more accurately depicting the dynamic migration features of topics in long-term texts [33], and can analyze text datasets with time attributes, identify their topics, and reveal the dynamic evolution of those topics [34]. Ha et al. [35] analyzed key factors influencing smartwatch sales using dynamic topic modeling on Reddit comment data. Bogdanowicz et al. [36] examined the dynamic trends of topics of public concern during the COVID-19 pandemic using Twitter text data. Shen et al. [37] analyzed evolving research trends and challenges in Alzheimer’s disease studies through dynamic topic modeling of historical literature. Nguyen et al. [38] identified factors influencing customer experience in the hotel industry using online review data, aiding businesses in improving products and services. Topic analysis of microblog texts using the DTM, as illustrated in Fig 2.

Here, *α*_*t*_ and *β*_*t*_ denote the Dirichlet prior parameters for text at time *t*, generated based on *α*_*t-1*_ and *β*_*t-1*_ respectively; *θ* follows a multinomial distribution with parameter *α*, defining the topic probability distribution for the document dataset, where topic *Z* is generated by multinomial distribution *θ*; *w* represents topic words selected by *β*, which defines the topic word probability distribution. As time progresses, the topic distribution parameter *α*_*t*_ at time *t* and the word distribution *βt,k* corresponding to topic *K* both evolve from their values at time *t-1*. Topic *K* at time *t* evolves smoothly from topic *K* at time *t-1*. Inputting the dataset into the DTM yields a time-topic matrix, a topic-time matrix, and a document-topic matrix. These matrices reveal the topic distribution across different periods, the evolution of each topic over time, and the topic distribution within each document.

**Fig 3:**
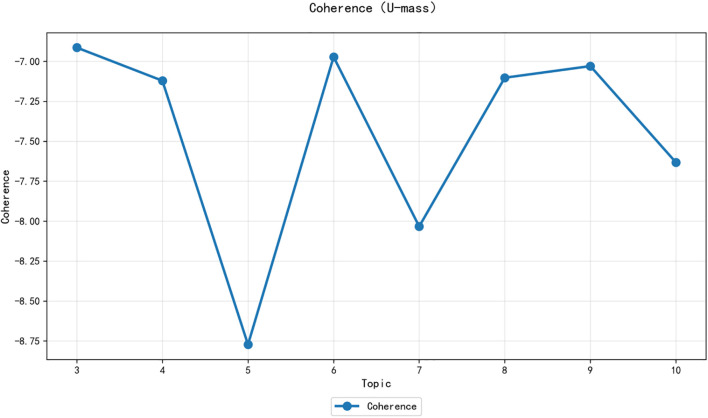
DTM topic-coherence change graph.

The result shown in Fig 3 coherence score is the highest within the interval when the number of topics is 6. Higher coherence scores indicated that the topic was cohesive, clear, and relevant, while lower scores suggested a lack of clarity, presence of noise, or irrelevance [39]. This paper selects 6 potential topics to analyze the attitudes of microblog users toward express services post-COVID-19.

#### CNN–LSTM.

Originally invented for computer vision, convolutional neural networks (CNN) [40] models have subsequently been shown to be effective for NLP and have achieved excellent results in semantic parsing [41]. However, CNN may fail to capture long-distance dependencies [42]. Long Short-Term Memory(LSTM) [43] method of deep learning, involves remembering the long distance relationships between the words which helps determine the sentiment of a sentence [44]. Therefore, this paper combines the CNN with LSTM to perform sentiment analysis on microblog texts. CNN is good for extracting large amounts of data and useful features using a convolutional architecture, whereas LSTM is better for extracting large amounts of data because of its recurrent architecture [45]. Zhang et al. [46] combined with the characteristics of Chinese grammatical structure, proposed a CNN-LSTM model for Chinese language fine-grained emotion computing. This paper combines DTM and CNN-LSTM models. In this way, the hot topics that different types of bloggers pay attention regarding express services can be effectively understood, and attitudes toward express services can be more effectively obtained, allowing an understanding of the public’s demands for express services post-COVID-19.

**Fig 4:**
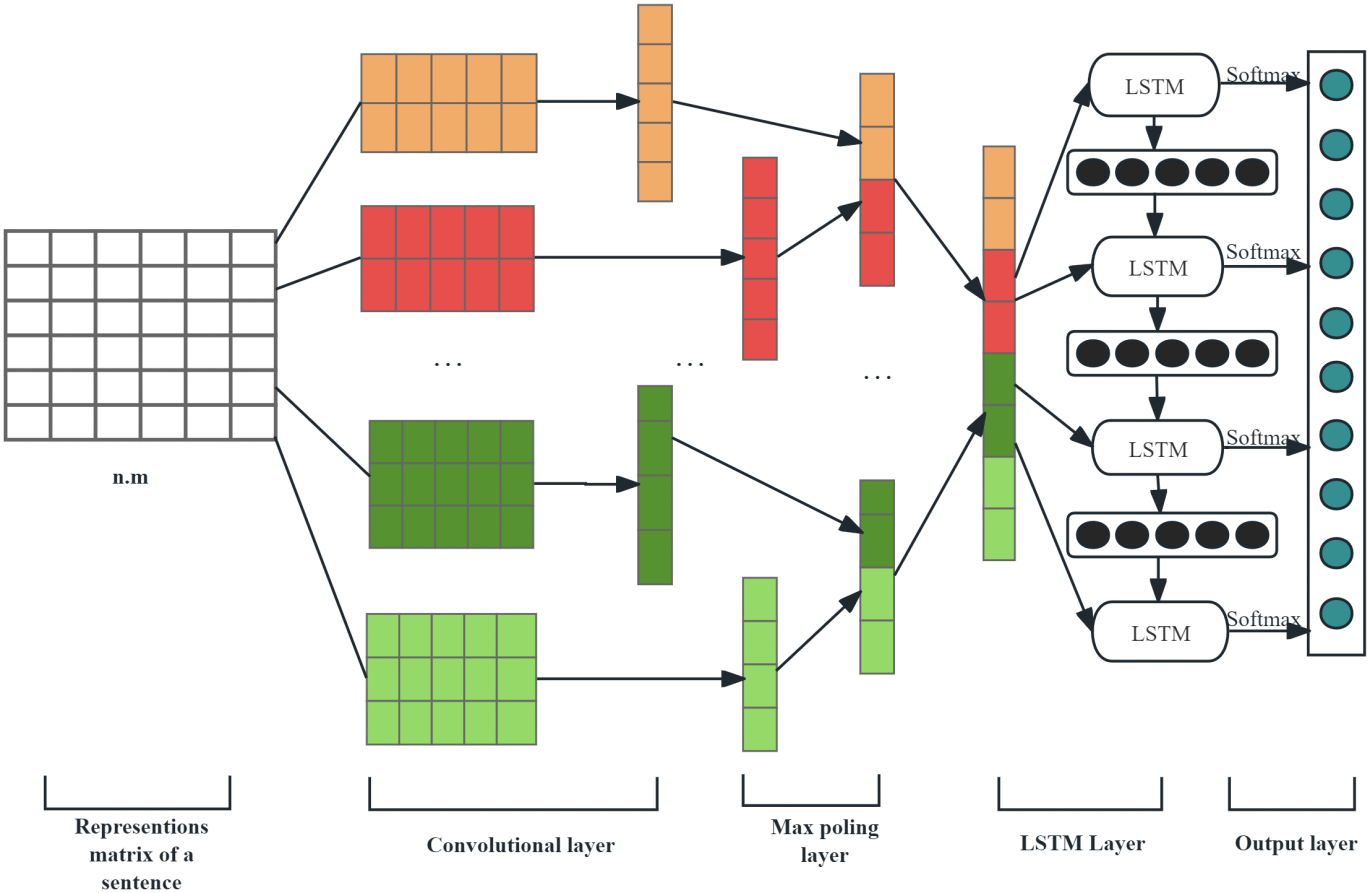
CNN–LSTM model structure diagram.

Fig 4 shows a diagram of the CNN-LSTM model structure diagram. This paper uses a combined CNN-LSTM model to perform sentiment analysis on microblog texts. The CNN can remove noise and consider the correlation between multivariate variables, and the LSTM models temporal information and maps time series into separable spaces to generate predictions [47]. The length of each microblog text is *n* in this paper, and *m* is the dimensionality of the word vectors. The two-dimensional word matrix *x* ∈ *R*^*n×m*^ is formed.

In this paper, three models are selected for comparison with CNN-LSTM: Bi-LSTM, GRU and CNN. A total of 19,000 microblog texts were selected with labeled emotions as the dataset for model training, with positive and negative comments each accounting for half. Eighty percent of the dataset is a training set, and the remaining 20% is a test set.Table 2 shows the parameter settings.

**Table 2:** Model parameters.

Batch size	128
**Epoch**	20
**Dropout**	0.5
**Hidden size**	512
**Filter numbers**	100
**LSTM layer numbers**	1

During the training process, microblog texts are preprocessed, text data are tokenized, four sentiment analysis models are constructed, and the optimal value of each model training is obtained. Dashtipour et al. [44] used precision, recall, F1-score, and accuracy to evaluate the performance of the proposed approaches.

**Fig 5:**
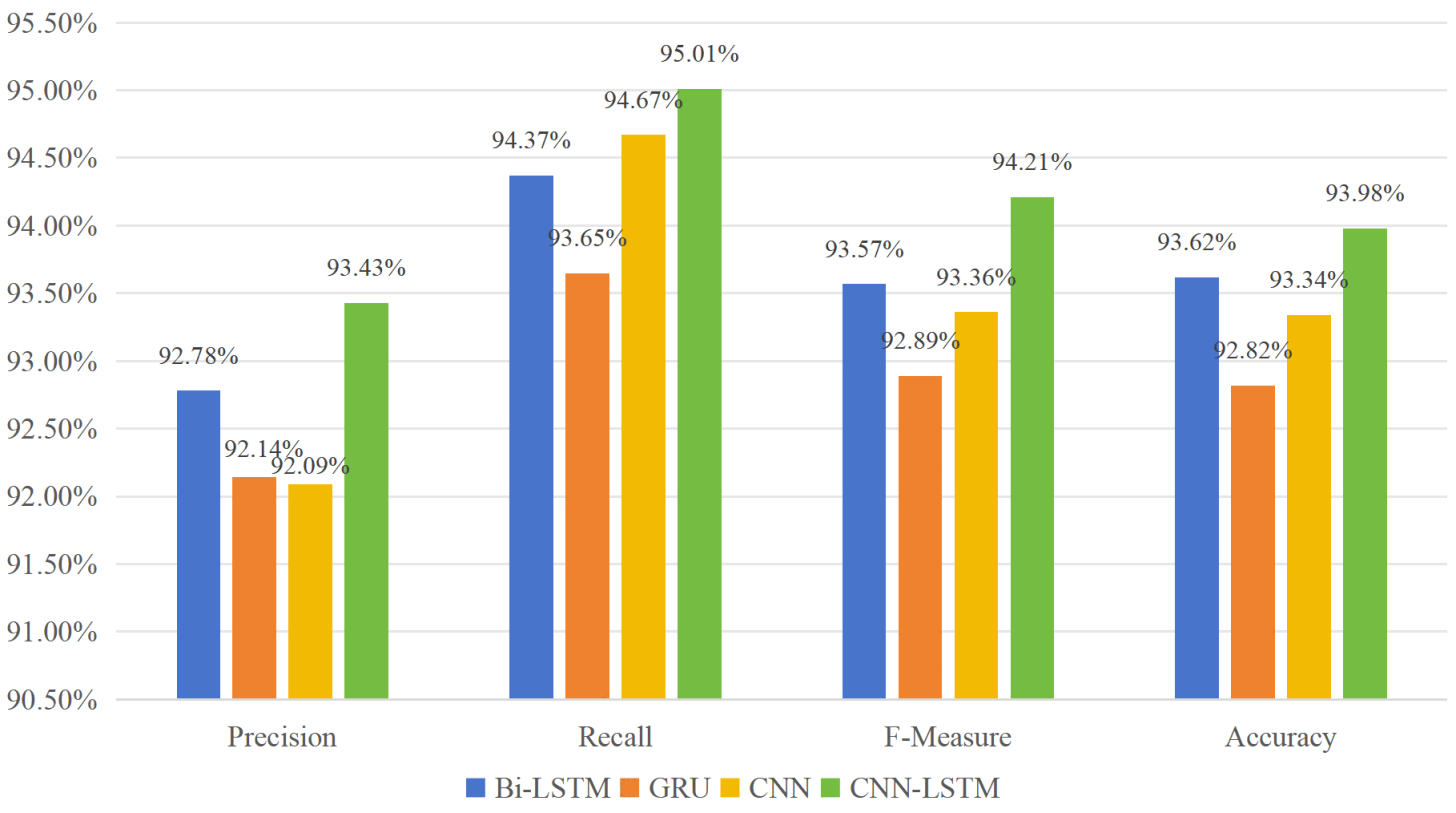
Comparison of model training results. The performance of Bi-LSTM, CRU, CNN, and CNN-LSTM is compared in terms of precision, recall, F-measure and accuracy, with CNN-LSTM achieving the best overall performance.

Fig 5 above, it can know that the CNN-LSTM model is superior to the other models in terms of precision, recall, F1-score, and accuracy, which shows that the CNN-LSTM model selected in this paper can better predict the sentimental tendency of microblog texts and that the public’s sentimental attitude toward express services can be accurately analyzed.

## Results

The captured content of each microblog text includes the user ID, whether the blogger is certified, the release time, the microblog text content,the forwarding volume,the comment volume,and the like volume. The types of blogger certification are divided into three categories: official, personal and noncertification. The microblog texts used are shown in Table 3.

**Table 3:** Data formats of the microblog texts.

Keywords	Blogger	Microblog certification	Blog	Release time	Retweets	Comments	Likes
Logistics	Beijing Traffic Broadcasting	Official	With the optimization and adjustment of China’s epidemic prevention policy, express and logistics has gradually resumed, and the volume of express shipments in Beijing has also increased to a certain extent. “Double Twelve” is coming soon. What kind of elimination measures have various express companies adopted? Epidemic prevention measures to deal with the “Double Twelve” express mail peak?	December 10, 2022 19:56	554	1573	16,603
Logistics	Boundless**	Personal	During the week, I bought the medicine, I could buy any kind of medicine in Beijing, not to mention that many of them were bought in some Taobao, but many of them were stuck in logistics.	December 12, 2022 18:31	73	352	7,053
Express	God**	Personal	JD.com has dispatched more than 1,000 couriers from all over the country to help Beijing.	December 14, 2022 20:56	43	200	1,649
Logistics	Qiuqiu Everything**	Noncertification	Single envelope:All have been sent out thus far, and the final batch number is not provided yet.	December 12, 2022 23:50	1	351	371

This paper focuses on microblog with official and personal certifications. Official certification has a certain popularity and influence.It is suitable for the official accounts of enterprises, governments, the media and other organizations. These accounts have a certain influence and contribute to society.The number of fans typically exceeds 10,000 and the degree of interaction is high. Microblog with personal certification are mostly ordinary personal media accounts of ordinary individuals. Neither the number of fans nor the number of followers is less than 50. The integrity of their accounts is relatively greater than that of ordinary users as they are recognized as real, active individuals. The bloggers with the two types of certification represent different types of bloggers, and the microblog texts they post are authentic and representative.

### Time dimension

#### Time trend of posting volume.

**Fig 6:**
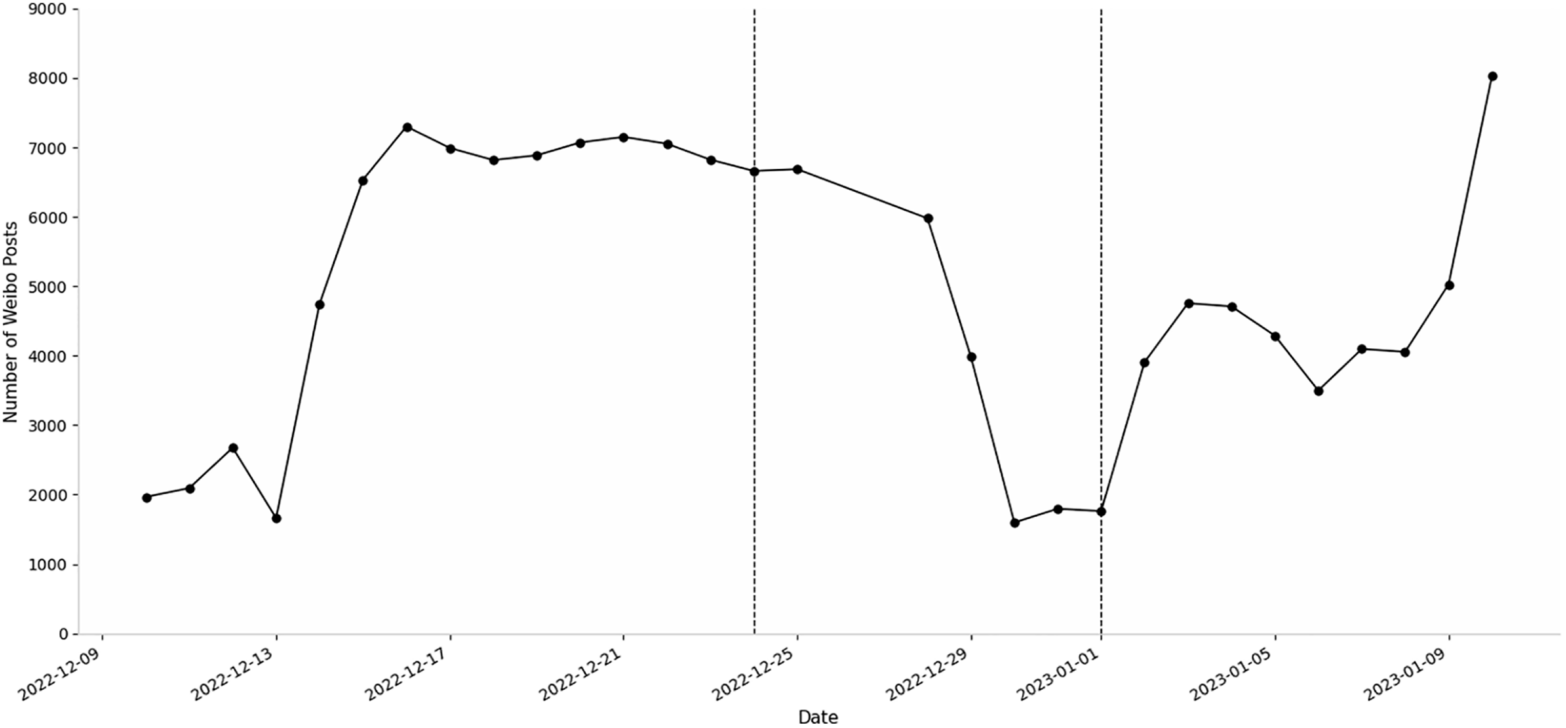
The daily posting volume of the all bloggers. Time series of daily Weibo posting volume from December 2022 to January 2023, showing two major peaks (December 10-23, 2022 and January 2-10, 2023) and a trough from December 24, 2022, to January 1, 2023.

Based on the trend of text volume changes over time, the evolution of online public opinion is divided into three development stages: December 10, 2022 to December 23, 2022; December 24, 2022 to January 1, 2023; and January 2, 2023 to January 10, 2023,as shown in Fig 6.

The peak period of the first phase of posting volume was from December 10 to December 23, 2022. The number of posts gradually reached its peak on December 15, and has remained stable at a high level ever since.With the arrival of the “Double Twelve” shopping festival, the demand for express packages increased sharply. According to data from the Ministry of Transport, the number of daily express deliveries across the country increased from a low of 250 million on November 28–340 million on December 15. With the gradual spread of the epidemic, there were courier shortages occurred during the peak period of infection in various regions, so the daily delivery volume nationwide from the 15th to the 18th continued to decline. During this period, a large-scale infection broke out after a seven-day incubation period. People from all walks of life were ill with COVID-19 and were staying at home. Since there was no way to go out to buy medicines, food or daily necessities, express delivery and takeaway became popular topics during this time.

A slump in posting volume occurred from December 24, 2022, to January 1, 2023. The posting volume of all the bloggers sharply declined. With the recovery of people infected in the first wave, the express industry gradually resumed normal operations. Since most people were under lockdown at home during the epidemic, people’s shopping methods changed after recovery, from online to offline consumption. Consequently, the demand for online express packages decreased, and the topic of express services decreased.

The second peak in posting volume occurred from January 2,2023, to January 10, 2023. Although the number of texts posted by bloggers peaked. According to data from the State Post Bureau, in early January, driven by favorable holiday consumption, the industry continued to operate stably, and the express delivery business volume (December 31, 2022 to January 2, 2023) increased by 15.2% compared with that of the previous year’s New Year’s holiday. On the 4th and 5th, the number of packages in the system exceeded 400 million for two consecutive days. The public’s attention to express delivery and logistics also rose rapidly; with the end of the festival, the public demand for express delivery decreased, and the public’s attention to express delivery gradually declined.

#### Time change analysis of the overall sentiment mean.

**Fig 7:**
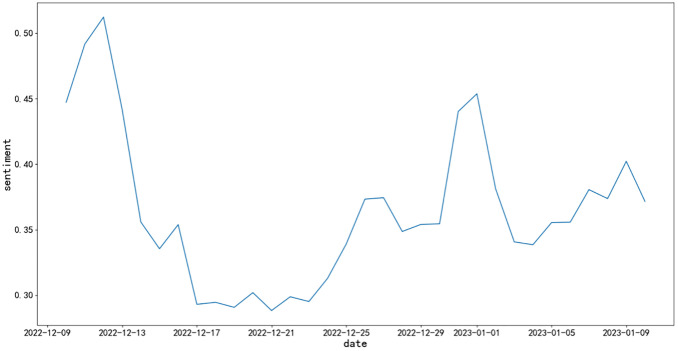
Time-varying graph of the average sentiment value of microblog texts. The sentiment value fluctuated within the range of 0.29-0.51, showing an overall negative trend, with two obvious emotional peaks corresponding to early December and around New Year’s Day respectively.

During the post-COVID-19 period, discussions of the epidemic and express on microblogs were very active. Sentiment analysis of microblog texts can explore the demands and expectations of the public for express services during an epidemic.The CNN-LSTM model converts the sentimental output into a digital form through the detach().numpy() function in order to obtain the specific emotional value. By calculating the daily average sentiment value, the sentiment average over time can be obtained, as shown in Fig 7.

Phase 1 (December 10, 2022 to December 23, 2022): Sentiment levels initially surged and then rapidly declined, with the number of posts reaching its first peak. The initial surge in posts likely included many expressions of anticipation for further easing of restrictions, resulting in a positive sentiment. On approximately December 12, 2022, the public’s sentimental value declined significantly. With the arrival of the Double Twelve shopping holiday, the volume of logistics and express services increased sharply. In addition, after the full lifting of COVID-19 restrictions, there was a shortage of personnel in the express service industry. The speed of express services did not meet the demands of the public, and dissatisfaction has risen.

Phase 2 (December 24, 2022 to January 1, 2023): Emotional value surged again, posting volume decreased, the public’s complaints about logistics during the epidemic had been largely released, and emotional expression entered a period of fatigue, so the overall posting volume naturally decreased. Logistics have gradually resumed, and some supplies have been delivered to meet the public’s needs.

Phase 3 (January 2, 2023 to January 10, 2023): The sentiment index dropped sharply on January 2nd, then gradually rose, and the number of posts also peaked. This period coincided with the New Year’s Day holiday (December 31, 2022 to January 2, 2023), coupled with the rapid increase in demand for New Year’s goods delivery, leading to an increase in express delivery volume. However, the on-duty rate of couriers had not yet recovered, resulting in problems such as backlog of packages, delivery delays, and unresponsive customer service.As the holiday effect gradually subsided and business volume returned to normal, the pressure on transportation capacity was further reduced. The proportion of packages actually received by users increased, service anomalies decreased, and satisfaction with express services rebounded, ultimately driving a gradual increase in emotional value.

### Attention dimension

#### Texts dimension.

This paper uses the EWM to calculate the information entropy and weight of the number of retweets, comments, and likes.According to the textual analysis, the smaller the information entropy of an indicator is smaller, the greater the amount of information provided by the indicator is greater, and the greater the degree of variability (variance) of the indicator is high. Therefore, the role it plays in the comprehensive evaluation should be greater, and the weight should be greater. Table 4 below shows a comparison of the information entropy and the weights of the three indicators.

**Table 4:** Information entropy and the weight of each index.

	Retweets	Comments	Likes
**Information entropy**	0.7530	0.8332	0.5997
**Weights**	0.3034	0.2049	0.4917

The information entropy of likes is the lowest (0.5997) and the weight is the highest (0.4917), indicating that the public’s attention to microblog texts related to express services is far more influenced by “likes” than “comments” and “retweets”. The information entropy of comments is the highest (0.8332) and the weight is the lowest (0.2049), indicating that a high number of comments alone does not mean high attention, and a comprehensive judgment needs to be made in combination with other indicators.

The differences in weights among retweets (weight 0.3034), comments (weight 0.2049), and likes (weight 0.4917) confirm that “a single metric alone cannot accurately measure public attention”—for example, if a microblog text receives a high number of comments but a low number of likes, it may be that the controversial content has sparked discussion rather than truly attracted attention.Therefore, the evaluation needs to combine multiple indicators.

To further verify the reliability of the weight of the number of likes, EWM is used to calculate the evaluation values of all the microblog texts,and obtain the top ten microblog texts with the highest evaluation values, as shown in Table 5 below:

**Table 5:** Top ten microblog texts sorted by the evaluation values.

Blogger	Microblog certification	Blog	Retweets	Comments	Likes	Evaluation value
Jiangnan**	Personal	Seen in Moments, China Express	9072	2199	87707	0.0207
Metro Times	Official	In Hechi, Guangxi, a girl stole parcels many times in obtain an Apple mobile phone, via “ Unboxing the Blind Box”. The police found that the girl could “swipe” 10 packages a day by riding a shared bicycle at her fastest. She stole so many packages that she couldn’t even remember. Because many parcels were of low value, buyers didn’t take it seriously when they couldn’t find them.	442	5,281	75,522	0.0138
Pear Video	Official	Weinan, Shaanxi. Mr. Sun’s sister-in-law sewed Merrill Lynch and other medicines into his clothes and sent them to him. Mr. Sun said that he had a child who needed medicine, but he couldn’t buy Merrill Lynch, and the hospital couldn’t order the drugs. His sister-in-law sent medicine to him. Mr. Sun said that because he saw on the internet that many people were not able to receive express delivery, to prevent this from happening, she sewed the medicine in his clothes and sent it safely, which resolved the urgent need.	1006	4,169	73,282	0.0136
West Decision	Official	Express Xiaoge Lost Gold Bowl Crying and Calling the Police: It’s worth 50,000 yuan, and the police checked and recovered it.	198	1,335	44,270	0.0077
West Decision	Official	#The man bought roses online and found a bullet after disassembling it#: Confiscated by the police, it has been filed and registered.In Hebei, a video of a man dismantling a package and finding a bullet was circulated online. Mr. Jiang, the party concerned, said that the video was shot before. At that time, he spent dozens of yuan to buy red roses online and found a bullet when he received the express delivery. After calling the police, the police came to collect the courier box and bullets.	385	2,513	48,704	0.0088
Boiling Point Video	Official	#Man cannot find his own package on the shelf and throws packages all on the ground# Post station boss friend: It took more than two hours to return. Surveillance showed that a man threw all the courier packages on the shelf on the ground just to find his own package. At that time, the man refused the help of the post station owner and insisted on finding it by himself. Later, the boss and the staff worked together for more than two hours to restore the packages to their original position. He hoped that the man could apologize to the station owner and employees.	566	2,958	47,945	0.0089
China News Weekly	Official	#Expert Analysis Beijing Current Infection Rate Not Reaching 70%# In the view of the aforementioned data experts, considering the pathogenicity of Omicron, the current infection rate in Beijing is less than 70% based on an estimate of 0.1% of severe cases. “Now the infected are mainly medical staff, express, young and middle-aged people, migrant workers, etc.” It will take some time before the peak of infection among the elderly will appear.	507	3,214	31,058	0.0061
Black Cat Complaint	Official	Man who bought fake Nike and reported shop was sent a wreath# A consumer in Chengdu complained to the platform that he bought a set of Nike sportswear online and took it to a physical store for appraisal. The clerk said that the item number of the clothes could not be found, and the quality and workmanship were not the same as the original. When the man communicated with the store, they also admitted that it was not genuine, so he applied for a return and reported the store on the platform, but then he received an express reminder from someone else who bought a wreath for him.	149	1,399	35,255	0.0062
Our**	Personal	First-Generation Express Courier Already Lived in Villa#. This is Zhongshan Township, Zhejiang. Many villas were built by first-generation courier. Tonglu, the county where he is located, is the hometown of express delivery in China, the birthplace of three links and one express delivery, supporting half of China’s express delivery industry. When we entered here, we discovered that there is no secret magic trick in the express delivery industry, only hard work. It is precisely because of the exquisite process and agile speed of Tonglu people whom we kept a 24-hour logistics record of on our mobile phone, that our consumption needs across the country can be met in three days.	6987	37	537	0.0046
Rb86-Northern**	Personal	From daily changes such as fewer takeaway riders and slower express delivery, we realize that we may have to go through a wave of difficult times after the release.	79	2,241	20,499	0.0039

Table 5 lists the top 10 microblog texts by evaluation value. The core findings focus on three types of highly-regarded content:

Courier group related content:Among personal certification bloggers, the blogger “Jiangnan**” (87,700 likes) has the highest evaluation value (0.0207). In addition to the text, there is a picture in the original microblog text that reflects the professional dedication of the JD courier; this has attracted great attention from the public.

Public welfare-related events: Of the ten microblog texts, seven are from official bloggers.Texts related to “drug delivery” (e.g., “Clothes sewn into medicine for delivery” in Weinan, Shaanxi, with 73,300 likes) and “package safety” (e.g., “Package theft for phone swap” in Hechi, Guangxi, with 75,500 likes) posted, reflecting that “content related to the vital interests of the public (health, property safety)” is more likely to attract high attention.

Content exposing service issues: Texts related to negative incidents such as “lost packages,” “violent throwing of packages,” and “bullets hidden in couriers” all made it into the top 10. Such content posted by official bloggers generally received a high number of likes (e.g., “Boiling Point Video” and “Western Decision”), reflecting the public’s awareness of supervision of express service issues and their recognition of official media’s exposure of such incidents.

#### Topic dimension.

**Fig 8:**
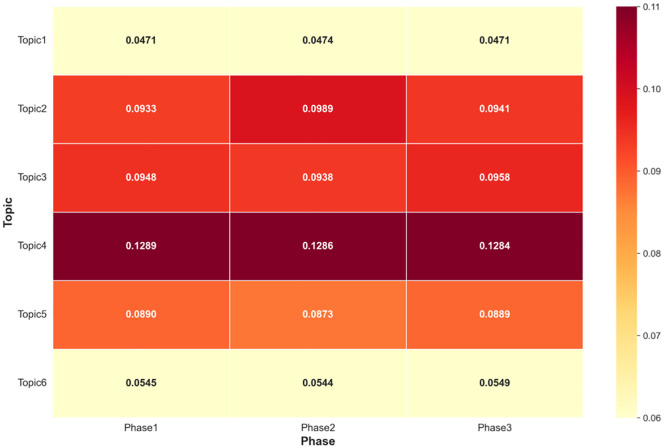
DTM_topic heat map(All).

This paper uses the DTM to conduct a topic analysis of social media posts related to express services from December 10, 2022 to January 10, 2023. Based on topic consistency scores, the optimal number of topics was determined, resulting in six core topic categories. Furthermore, ten keywords with high weight and strong representativeness were selected from each topic to understand the specific focus of public attention on express delivery services after changes in epidemic prevention and control policies.The results are shown in Table 6 and Fig 8.

**Table 6:** All bloggers topics and word distribution.

Number	Topic Name	Keywords
Topic1	Daily Life and Health Status	fever, go home, things, night, go out, come back, hope, receive, finally, find
Topic2	Good safety	package, man, find, police officer, lost, online shopping, video, boss, netizen, western
Topic3	Courier Rights	Beijing, courier, work, company, guarantee, logistics, delivery guy, COVID-19, postal, service
Topic4	After-sales service	JD.com, logistics, shipping, customer service, SF Express, express delivery, courier, complaint, phone call, receive
Topic5	COVID-19 Prevention and Medication	mask, infection, antigen, ibuprofen, COVID-19, coronavirus, disinfection,relaxation, fever, hospital
Topic6	Governments’ requirements for enterprises	logistics, company, development, China, enterprise, service, market, growth, e-commerce, economy

Combining the topics of content and heat map, it can be concluded that good safety, courier rights, and after-sales service are the hot topics of public’ attention, and they have always been the focus of public opinion in all three stages, indicating that service experience is an important factor affecting the public’s attitude towards express services.

Topic 4 maintained high popularity throughout all three stages, highlighting the direct impact of service quality on public attitudes. The keyword distribution shows a concentration of high-frequency terms such as JD.com, SF Express, shipping, customer service, complaints, and telephone, directly reflecting significant public dissatisfaction with the service efficiency and experience of mainstream express delivery companies.Topic 2 showed high fluctuations, with a significant increase in popularity during the second phase. The keyword “lost” reflects the phenomenon of lost packages. The third most watched post in the attention dimension mentioned the phenomenon of stolen valuables. The main reason is that the overlap of Christmas and New Year’s Day caused a surge in logistics demand, resulting in a large backlog of parcels, a significant drop in the efficiency of the delivery system, and insufficient response from regular complaint feedback channels, leading to a concentration of various service conflicts.The popularity of Topic 3 is gradually rising, with the keyword “guarantee” pointing to the issue of safeguarding the rights and occupational protection of delivery workers, reflecting the public’s concern for the protection of the rights and interests of workers.

(1) Comparative analysis of topics of attention in different periods of COVID-19

Before and after the COVID-19 outbreak, many scholars studied the public’s attention to express services from online reviews. Table 7 shows the meaning of the topics in the field of express services in different periods of COVID-19.

**Table 7:** Topics of attention in different periods of the COVID-19.

Period	Time	Topics of attention	Meaning	Document source
pre-COVID-19	2015–2017	Customer service	Refers to the manual consulting service provided by the enterprises.	Mining Express Service Innovation Opportunity From Online Reviews
		Service attitude	Refers to the service attitude of employees towards customers when providing services.	
		Contact channel	Indicates whether the channels between customers and employees are smooth.	
		Goods safety	Indicates whether the goods arrive at customers safely and completely.	
		Information tracking	Represents the service that enables customers to query the location information of the express service in real time.	
	2019	Logistics speed	Represents express delivery speed or slow.	Research on Customer Satisfaction of Express Service Based on Text Mining
			Service attitude	Indicates the service situation of customer service and courier in the express services.
			Telephone service	Indicates how much customers value telephone service in express delivery services.
			Complaint handling	Refers to the handling of customer complaints.
			Logistics information update	Refers to express information update in transportation.
			Timeliness	Indicates whether the express can arrive within the agreed time, including the timeliness of shipment, delivery,and transit.
			Charging price	Refers to the price of express services.
during COVID-19	2021	Delivery time commitment	Indicates the commitment to the delivery timeline from the shipment’s origin to its final destination.	The main influencing factors of customer satisfaction and loyalty in city express delivery
		Matching degree between price and service	Indicates the express service price should match the service quality.	
		Popularity and credibility	Refers to the popularity and reputation of express delivery enterprises.	
		Mailing security	Indicates whether the express can safely reach the customer.	
	2022	Goods safety	Indicates the safety of the express itself in the process of express service, such as the express package without damage, the interior is complete, and the function is intact.	Research on improving express service quality based on online review data mining
			Timeliness	Indicates whether express delivery can be timely and accurate.
			Service attitude	Indicates express staff (customer service, Courier) in contact with consumers (door-to-door delivery, telephone communication) there are rude words and deeds, bad attitude and other courtesy problems.
			After-sales service	Indicates express staff handle consumer complaints (such as lost items, damage compensation, etc.) is not timely, consumer complaint channels are not smooth, and the complaint process is complicated and cumbersome.
post-COVID-19	2023	Daily Life and Health Status	Daily scenarios around fever, going out and returning home	this paper
		Good safety	Social news involving lost packages and police intervention	
		Courier Rights	Focus on labor protection of delivery workers during the pandemic	
		After-sales service	Consumer issues regarding shipping, customer service complaints	
		COVID-19 Prevention and Medication	Pandemic-related topics including masks, antigen tests, fever reducers	
		Governments’ requirements for enterprises	Macro-level logistics market and e-commerce economy	

Before the outbreak of COVID-19, “customer service”, “service attitude”, “telephone service” and “complaint handling” indicated that the public paid attention to after-sales services. “Service attitude” and “logistics information” were also hot topics of the public’s attention. During the COVID-19 pandemic, the public has paid more attention to delivery time and goods safety. According to the research results of this paper, compared with before and during the COVID-19 outbreak, the after-sales service, and good safety after the full lifting of COVID-19 restrictions still received more attention from the public than they did before and during the COVID-19 outbreak. Moreover, after the full lifting of the COVID-19 restrictions, more attention was given to couriers and governments’ requirements for enterprises.

(2) Analysis of official certification and personal certification topics

**Fig 9:**
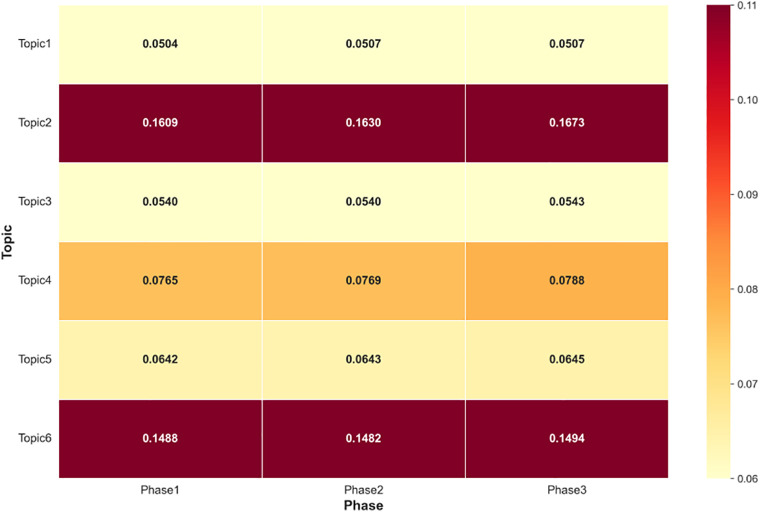
DTM_topic heat map(Official).

**Fig 10:**
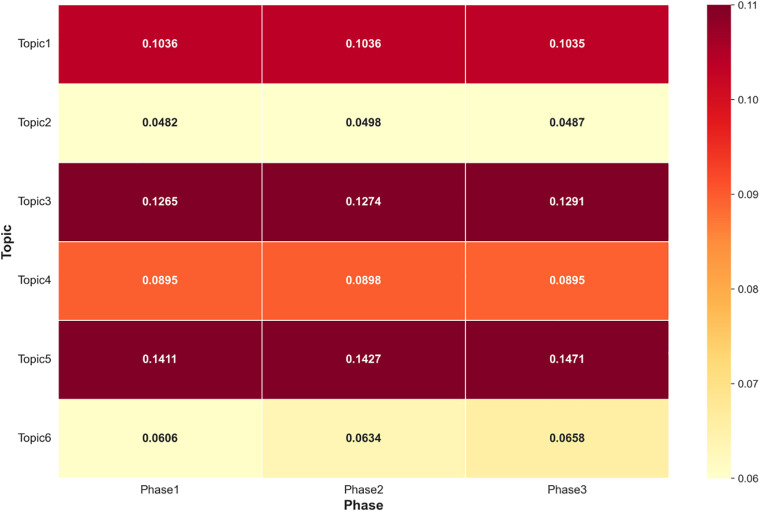
DTM_topic heat map(Personal).

Owing to the different backgrounds and positions of official and personal certification bloggers on microblogs, their views on express services may vary. Official certified bloggers, who often represent entities with commercial interests or public responsibilities, may focus on the performance of express services in response to emergencies or special periods. In contrast, bloggers with personal certification tend to represent individual perspectives and experiences, and their views on express services may be more diverse and personalized. Understanding the views of different types of bloggers can provide a more comprehensive understanding of various aspects of express services, which can help the industry make more informed choices and evaluations. Tables 8 and 9 shows the different certification types of blogger topics and word distributions. Figs 9 and 10 show the topic heat map for official bloggers and personal bloggers, respectively.

**Table 8:** The official certification types of the blogger topics and word distribution.

Number	Topic Name	Keywords
Topic1	Parcel Receiving Experience	things, received, express delivery, finally, go home, feeling, shipping, come back, hope, find
Topic2	Express Enterprises	JD.com, logistics, customer service, SF Express, shipping, ZTO Express, complaint, YTO Express, express delivery, package
Topic3	Logistics Macro Economy	China, company, logistics, economy, technology, stock, international, market, plate, development
Topic4	Service expectations	enterprise, service, logistics, epidemic, work, guarantee, postal, personnel, prevention and control, supplies
Topic5	Epidemic Prevention Life	food delivery, mask, go out, fever, feeling, home, infection, disinfection, evening, relaxation
Topic6	Courier Rights	courier, Beijing, Cainiao, delivery guy, package, door-to-door delivery, support, reinforcement, subsidy, one after another

**Table 9:** The personal certification types of the blogger topics and word distribution.

Number	Topic Name	Keywords
Topic1	Timeliness	shipping, JD.com, received, SF Express, logistics, customer service, merchant, transit station, address, epidemic
Topic2	Personal Life	ibuprofen, go out, package, disinfection, feeling, friend, home, clothes, find, go home
Topic3	After-sales service	courier, express delivery, YTO Express, phone call, ZTO Express, complaint, make a call, customer service, delivery, stations
Topic4	Infection and Medical Care	infection, epidemic, COVID-19, mask, relaxation, hospital, positive, courier, nucleic acid test, health
Topic5	Courier Rights	courier, Beijing, Cainiao, delivery guy, food delivery, subsidy, postal, guarantee, Spring Festival, work
Topic6	Stock Market Trends	shipping, JD.com, received, SF Express, logistics, customer service, merchant, transit station, address, epidemic

Judging from the official blogger’s topic table and heat map, Topic 2 shows a steady upward trend and remains dominant. The high popularity and continuous growth of this topic, with express delivery companies such as JD.com, SF Express, ZTO Express, and YTO Express being frequently mentioned, reflects that the official blogger regards the service quality of leading companies as a core focus of supervision. Topic 6 remains stable at a high level. This topic indicates that Beijing, as the capital, is focusing its publicity efforts on measures to ensure courier are supported, subsidies are provided, and home delivery services are offered. Topic 4 continues to rise in popularity, becoming the third most popular topic. This topic focuses on enterprise-level epidemic prevention and control, material supply, and personnel management. Its significant rise in Phase 3 reflects the continued focus on enterprises’ ability to maintain normalized epidemic prevention measures in the post-pandemic era.

Judging from the topic tables and heat map of personal certification bloggers, Topic 5 has consistently ranked first and continues to rise, reflecting the high level of attention that individual bloggers pay to the deliveryman group. Topic 3 focuses on keywords such as delivery personnel, complaints, customer service, delivery, and stations, reflecting the problem of insufficient supply and demand matching after service recovery. Official certification bloggers focus on the service quality of brand companies, while individuals directly address the conflicts in last-mile delivery. Topic 1 is the fundamental focus throughout, with keywords such as “JD.com, SF Express, and logistics” reflecting the urgent need for express delivery companies to “deliver goods and supplies in a timely manner,” which was a core public concern in the early stages of the pandemic.

## Conclusions

### Main findings

This paper, through multi-dimensional analysis of Weibo data from December 10, 2022 to January 10, 2023 (the first month after the full lifting of COVID-19 restrictions policies), reveals new trends in public’s attention to express services, sentimental attitudes, and cognitive differences among different communication subjects in the post-epidemic era, and yields the following findings:

First, public sentiment was generally negative. The CNN-LSTM sentiment analysis showed that the average sentiment score for express services during the study period was below 0.5, indicating an overall negative bias. This sentiment fluctuation was highly correlated with logistical stress events (the “Double Twelve” shopping festival, the peak of the COVID-19 outbreak, and the New Year’s Day holiday), suggesting that the structural contradiction between service supply capacity and sudden surges in demand was the dominant factor causing public dissatisfaction. Although pandemic control measures have been relaxed, the recovery of the express delivery system lagged behind public expectations, leading to a continued accumulation of negative emotions.

Second, the core attention showed stability. The DTM identified six core topics, among which “good safety” and “after-sales service” were the core public’s attention throughout all three phases, consistent with the research findings before and during the pandemic, reflecting the importance of the fundamental dimensions of service quality. Li et al. [48] showed that in the early stages of the 2020 pandemic, the number of users dissatisfied with “logistics guarantee” was the highest, reflecting that during the peak of the pandemic, users were more eager for the safe and timely delivery of goods and supplies. The reasons affecting good safety include inadequate regulations and systems, or insufficient staff training. Often, courier picking up packages do not carefully verify the customer’s identity or thoroughly inspect the integrity of the goods [49]. Yan HQ [50] revealed that the problems with the good safety are caused by intense market competition, inadequate market supervision, and poor corporate management. Ding P [51] indicated that inadequate after-sales service refers to issues such as untimely handling of consumer complaints by courier staff (e.g., lost or damaged items, compensation), obstructed channels for consumer complaints, and complex and cumbersome appeal processes.

Third, the public’s attention has revealed two emerging trends: courier rights and governments’ requirements for enterprises. Pre-COVID-19 research mainly focused on functional service attributes (customer service, contact channels, information tracking), while research during the pandemic emphasized operational resilience (the match between delivery time, price and service). The findings of this paper demonstrate the maturity of public discourse—an increased systematic focus on labor welfare and institutional governance. The topic of “courier rights” indicates that, after the pandemic, public’s attention has become more comprehensive, encompassing both immediate demands for the efficiency and quality of delivery services and long-term concerns about the living conditions and rights protection of couriers [52]. The “Opinions on Doing a Good Job in Protecting the Legitimate Rights and Interests of Courier” issued in 2021 pays more attention to the rights and interests of courier, and this study confirms that this policy concern has been internalized as a component of public value judgments. Topic such as “governments’ requirements for enterprises” suggests that the public expects the government to play a more active regulatory and guiding role to promote corporate social responsibility and ensure the healthy development of the industry.

Fourth, significant differences exist in the perspectives of different communication entities. Official certification bloggers, as architects of institutional significance, focus on macro-level industry performance and policy compliance, emphasizing the service quality of “express enterprises”, “logistics macro economy”, and “service expectations” and assume the functions of industry supervision and policy communication. Personal certification bloggers act as experience sensors, focusing on service experiences and consumer rights at the micro level. They pay attention to specific scenarios such as “timeliness”, “personal life experience”, and “infection and medical care”, directly reflecting conflicts in end-services and insufficient supply-demand matching.It is worth noting that the two groups reached a consensus on the issue of “courier rights”, jointly promoting attention to workers in new employment forms. This differentiation aligns with the “structural hole” [53] theory in social networks—different types of actors occupy heterogeneous information niches, achieving complementarity and integration of market intelligence by bridging structural holes.

### Theoretical implications

Based on the above findings, this study makes the following theoretical contributions to existing literature:

First, it expands the application boundaries of the DTM in express services research. This paper introduces the DTM into post-crisis public service quality research, revealing the temporal heterogeneity of service quality perception by capturing the dynamic evolution of public concerns during critical transition periods. Compared to the LDA model, the DTM can identify the phased fluctuations in topic intensity and the semantic drift of topic content, providing a more detailed analytical tool for understanding the recovery patterns of service systems after crises.

Second, the effectiveness of the CNN-LSTM hybrid model in sentiment analysis of Chinese social media was verified. The model’s superior performance compared to Bi-LSTM, GRU, and CNN (leading in precision, recall, F1 score, and accuracy) demonstrates its suitability for the short, informal, and context-dependent text features of the Weibo platform. This methodological advancement provides a replicable technical framework for subsequent research on public sentiment towards the service industry.

Third, this study advances the theoretical understanding of stakeholder perspective differences in service evaluation. It reveals the functional differentiation between official certification bloggers and personal certification bloggers in the information ecosystem, enriching the research on the micro-mechanisms of multi-stakeholder service evaluation in the platform economy era.

Fourth, it expands the explanatory boundaries of the Service-Dominated Logic (S-D Logic). The topic of “courier rights” indicates that in platform-mediated service ecosystems, consumers increasingly base the legitimacy of value co-creation on the fair treatment of all participants, rather than focusing solely on maximizing the use value for end users [54]. This finding responds to research on labor rights in the gig economy and provides a new empirical foundation for understanding value ethics in service ecosystems.

### Management implications

#### Management suggestions for express enterprises.

Operational Dimension: Strengthen the elastic supply capacity for peak demand. Enterprises should establish a linkage mechanism with the local labor market, reserve temporary couriers to cope with personnel shortages during the epidemic and shopping festivals; use big data to predict peak order volumes during “Double Twelve” and New Year’s Day, deploy transportation capacity in advance, add regional transfer centers, and lease temporary transport vehicles to reduce package backlog; install surveillance cameras in the sorting and transportation process to capture and hold accountable those responsible for rough handling in real time.

Governance Dimension: Transforming worker protection into brand differentiation assets. Providing courier with accident and critical illness insurance, and setting up rest stations with drinking water and charging facilities to enhance the attractiveness of the job; linking cargo safety and customer communication training to performance to improve professionalism; and drawing on JD.com’s practice of paying social security for delivery personnel to transform rights protection into brand trust capital.

Service dimension: Optimize after-sales response mechanism. Establish a 24-hour online customer service channel, promising “responding to complaints within 1 hour and providing solutions within 24 hours,” and publicly disclose the progress of complaint handling to enhance user trust; implement recipient identity verification to prevent packages from being misdelivered or stolen.

#### Policy recommendations for governments and regulators.

Institutional supply: Improve industry standards and emergency response protocols. Require enterprises to develop emergency plans (including human resource reserves and transportation capacity allocation plans) for special circumstances such as epidemics and extreme weather, and report them to regulatory authorities regularly; clarify the calculation standards for lost or damaged packages (based on the actual value of the goods or a multiple of the express delivery fee) to reduce disputes between companies and consumers.

Regulatory Mechanism: Establish a quantitative rating and incentive system. Indicators such as on-time delivery rate, complaint rate, and cargo safety incident rate will be incorporated into the enterprise rating system and regularly publicized to guide consumer choices. Enterprises that implement measures to protect couriers and maintain emergency reserves will be given incentives such as tax breaks, subsidies for station construction, and training subsidies to reduce operating costs and encourage the fulfillment of social responsibility.

Collaborative governance: Establishing cross-departmental cooperation channels. Jointly with transportation, health, and other departments to establish “green channels” for delivery vehicles to ensure smooth logistics; monitor the coupling between social media sentiment and policy agendas, using public perception as a real-time input for policy effectiveness evaluation.

#### Limitations and future research trends.

First, this paper relies on Weibo and excludes users from other platforms (Douyin, Xiaohongshu), potentially introducing demographic bias. Second, while the one-month observation window captures the immediate effects of policy transitions, it may not fully represent long-term evolution of public attention. Third, subtle differences in the Chinese language (puns, irony, context-dependent expressions) may challenge the accuracy of automated sentiment classification.

Future research should extend the time frame to examine whether emerging themes (courier rights, governments’ requirements) persist or diminish as normalization progresses. Comparative studies in a cross-national context can assess whether China’s public attention patterns are applicable to other markets.

## Supporting information

S1 File S1_raw_images. (PDF)
