# Supplementary material for: Will there be new trends in the public’s attention to express services in the post-COVID-19 era?
Source: PLoS One. 2026 Jun 24;21(6):e0348096. doi: 10.1371/journal.pone.0348096 (PMC13293422; doi:10.1371/journal.pone.0348096)
Supplement: S1 File — (PDF) [file pone.0348096.s001.pdf]

PNG

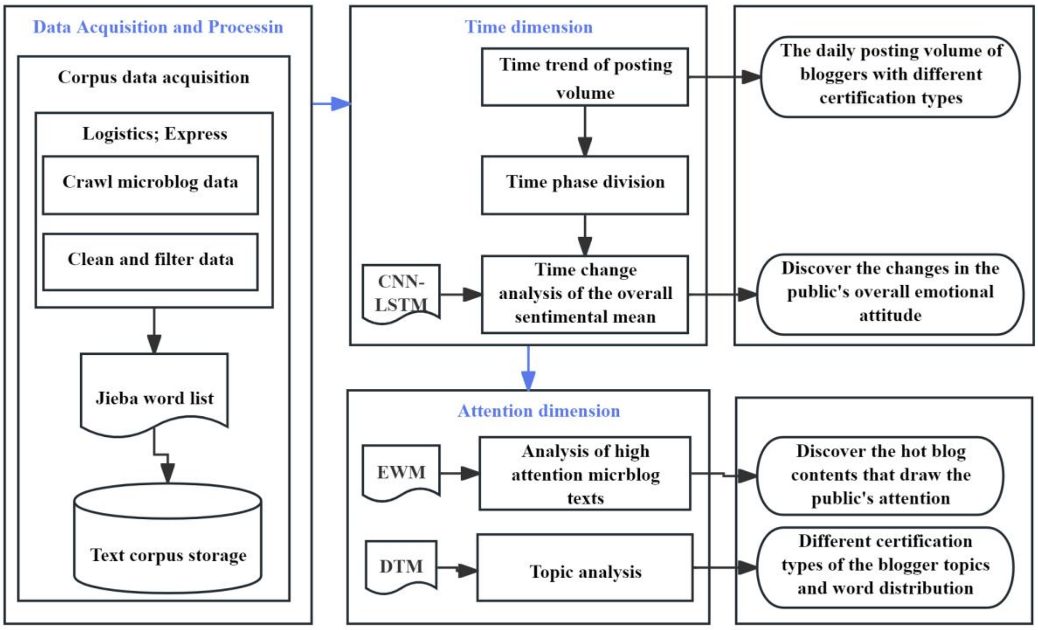

Fig 1. the overall theoretical framework.

PNG

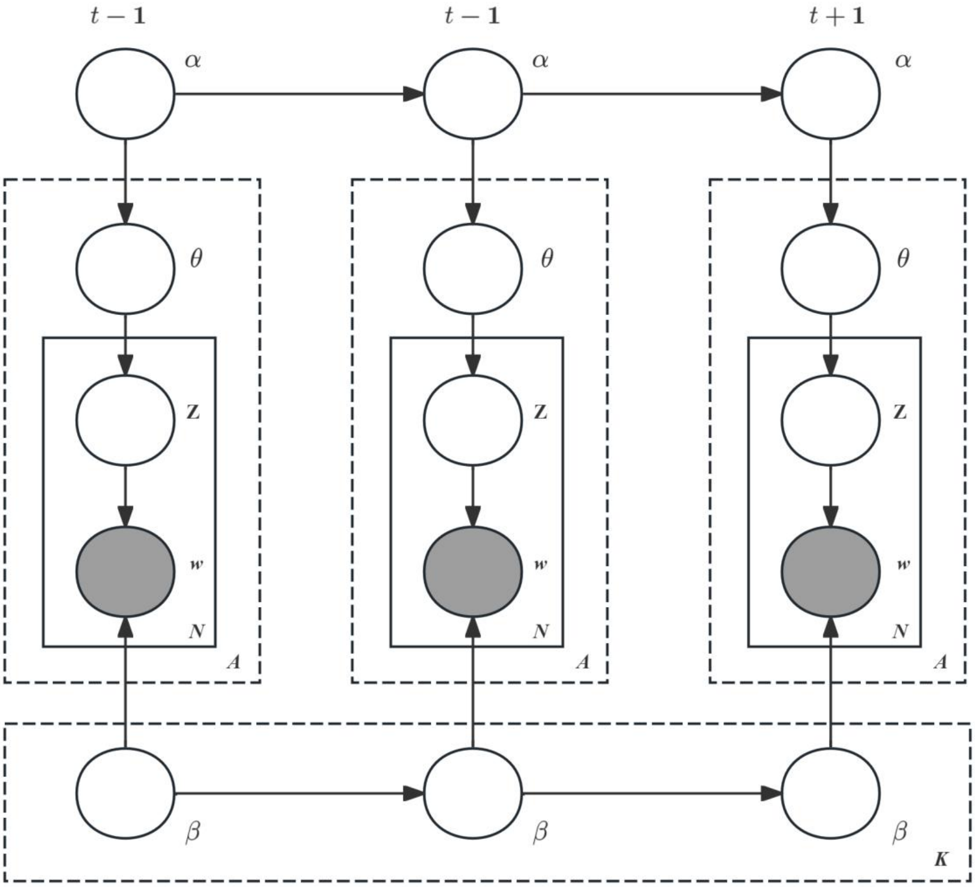

Fig 2. Dynamic topic modeling.

PNG

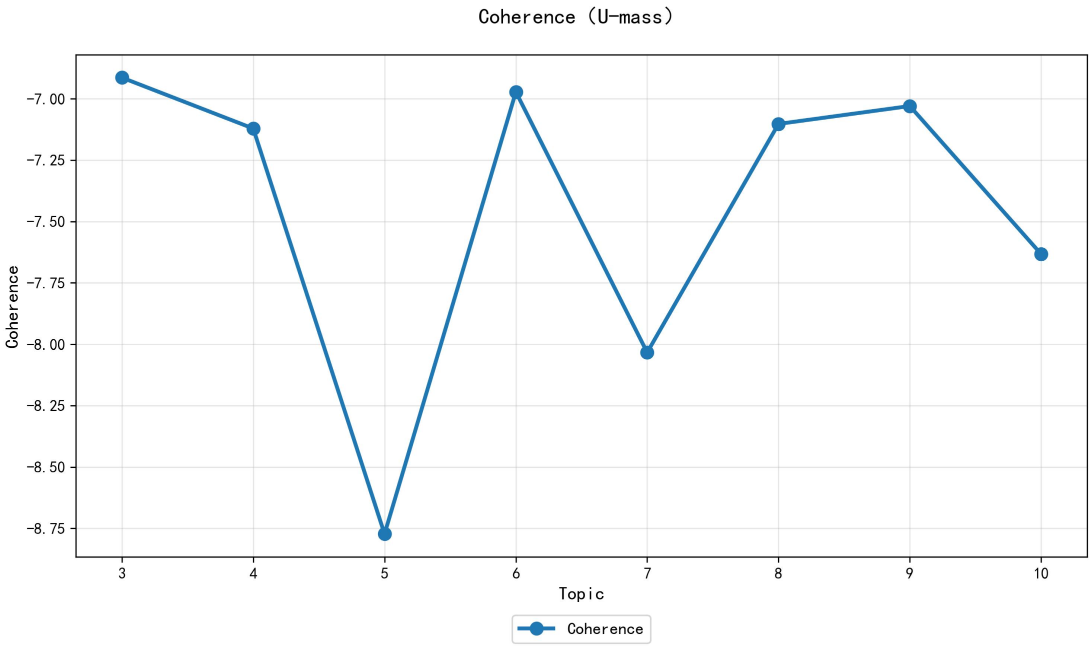

Fig 3. DTM topic-coherence change graph.

PNG

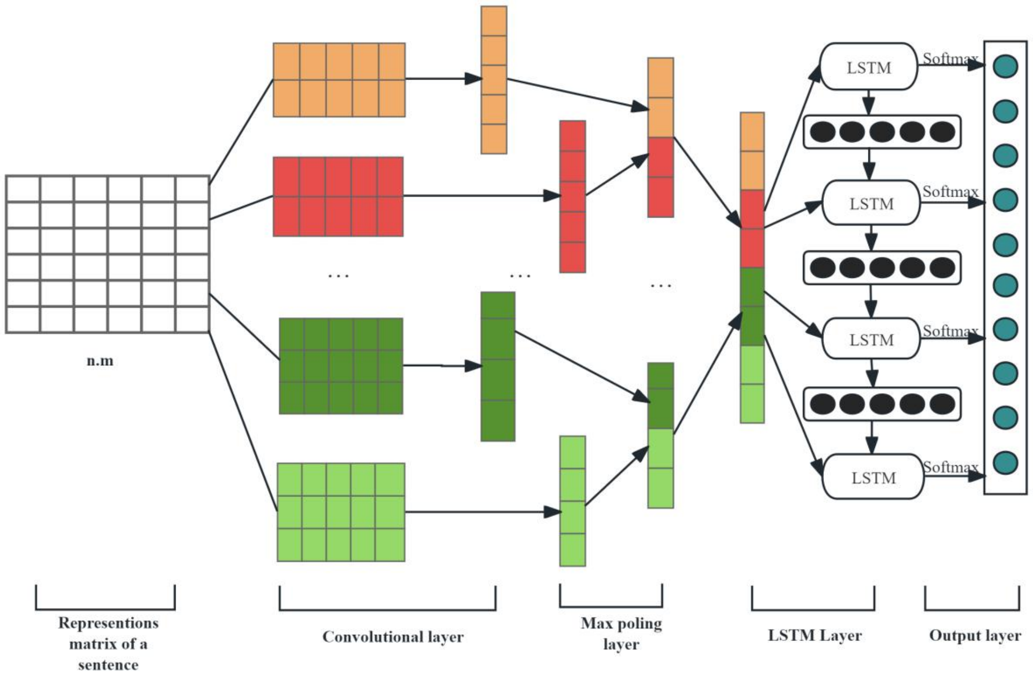

Fig 4. CNN–LSTM model structure diagram.

PNG

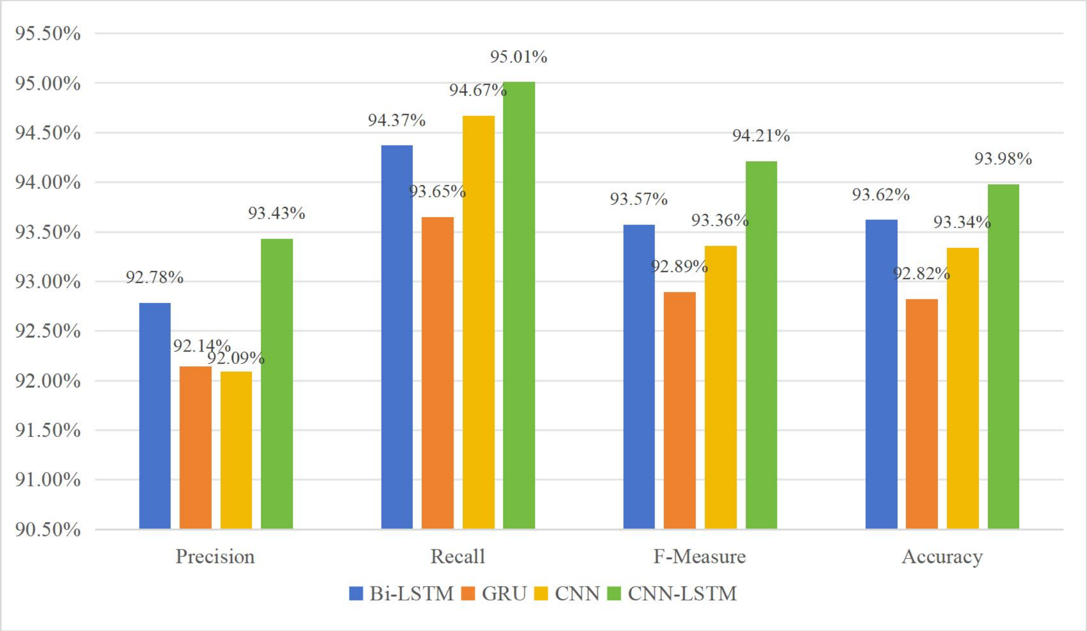

Fig 5. Comparison of model training results. The performance of Bi-LSTM, CRU, CNN, and CNN-LSTM is compared in terms of precision, recall, F-measure and accuracy, with CNN-LSTM achieving the best overall performance.

PNG

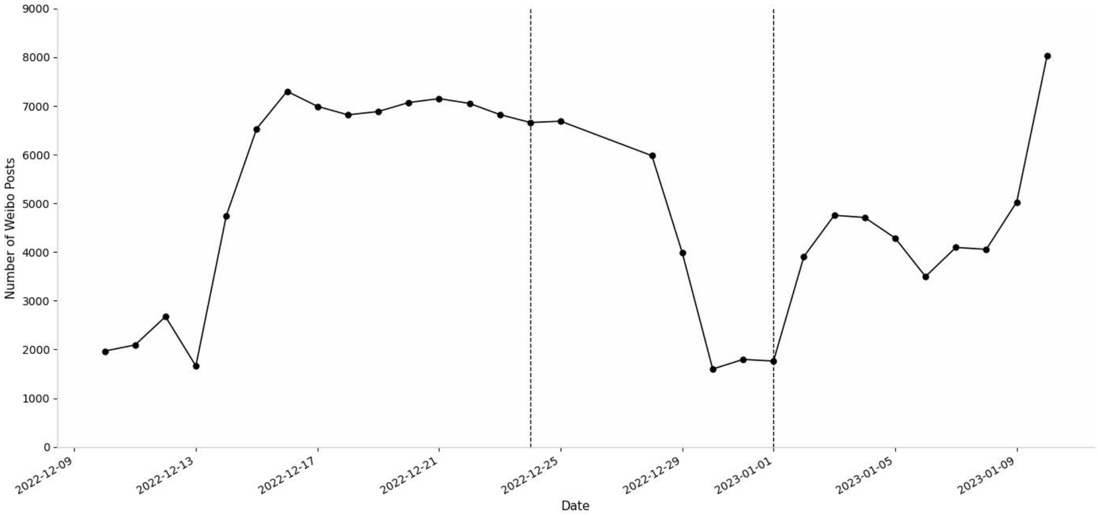

Fig 6. The daily posting volume of the all bloggers. Time series of daily Weibo posting volume from December 2022 to January 2023, showing two major peaks (December 10-23, 2022 and January 2-10, 2023) and a trough from December 24, 2022, to January 1, 2023.

PNG

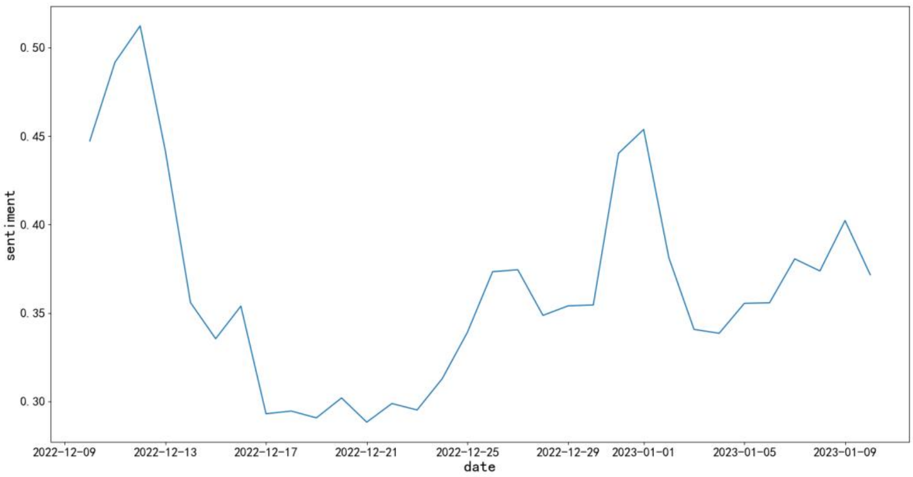

Fig 7. Time-varying graph of the average sentiment value of microblog texts. The sentiment value fluctuated within the range of 0.29-0.51, showing an overall negative trend, with two obvious emotional peaks corresponding to early December and around New Year's Day respectively.

PNG

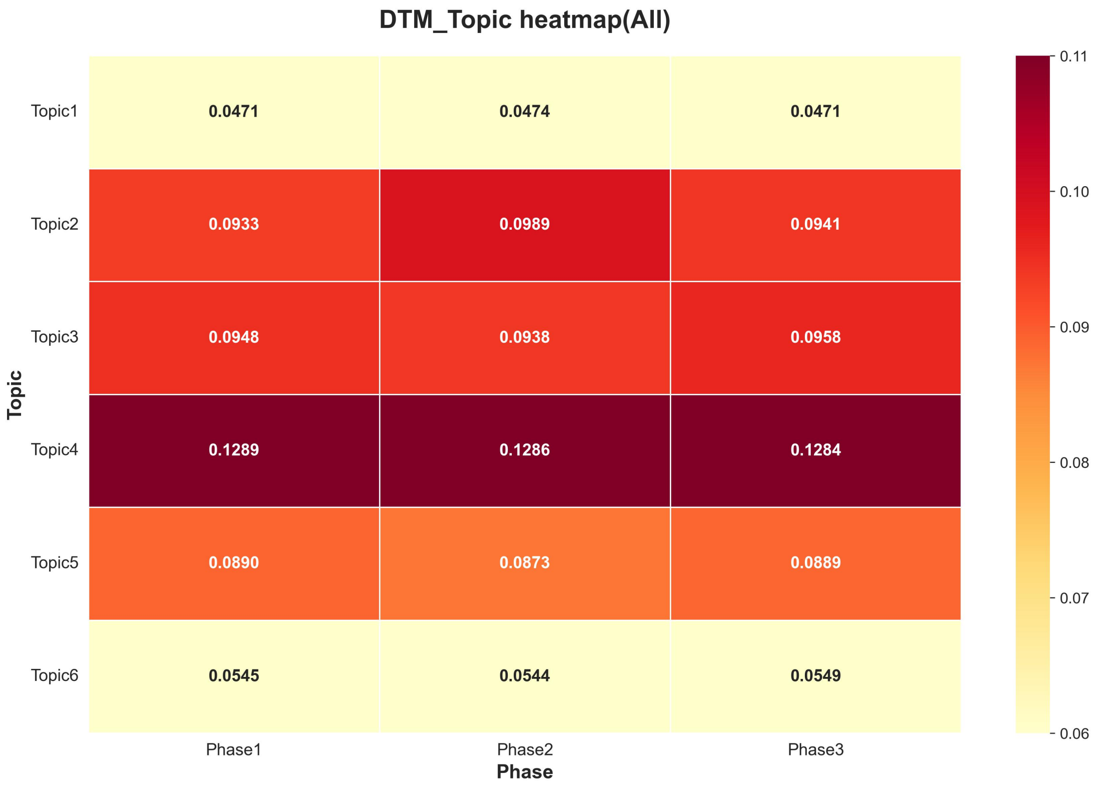

Fig 8. DTM_topic heat map(All).

PNG

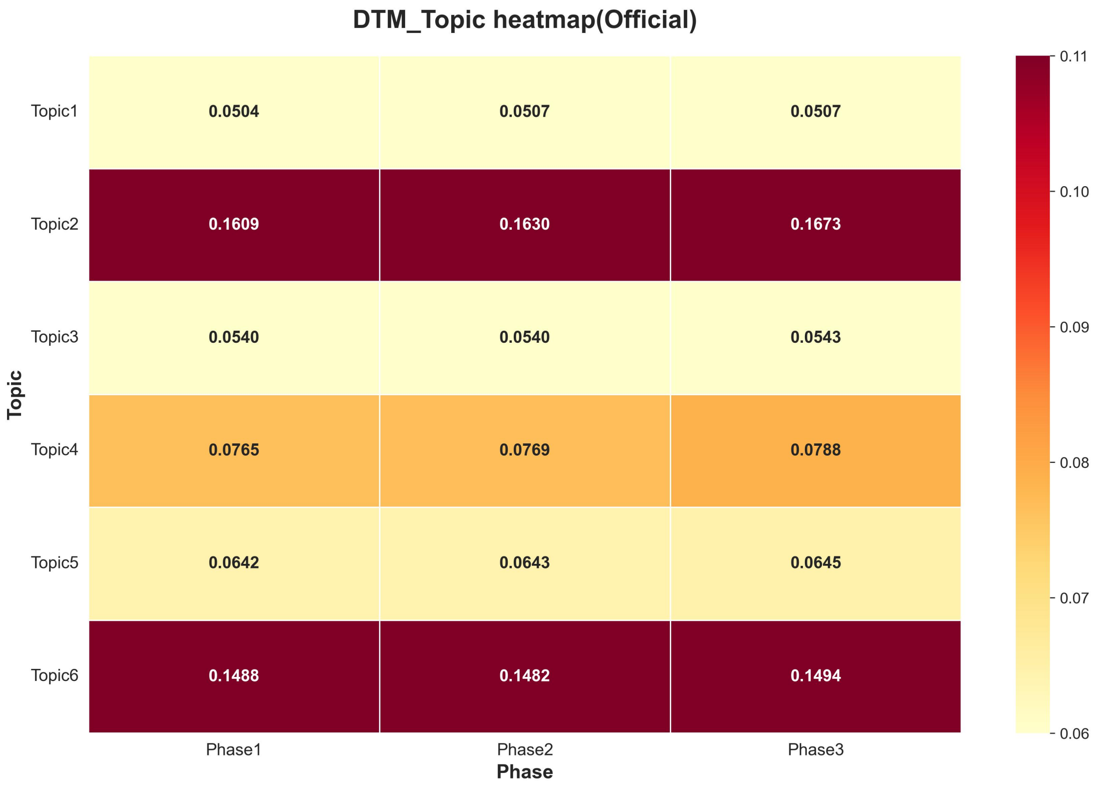

Fig 9. DTM_topic heat map(Official).

PNG

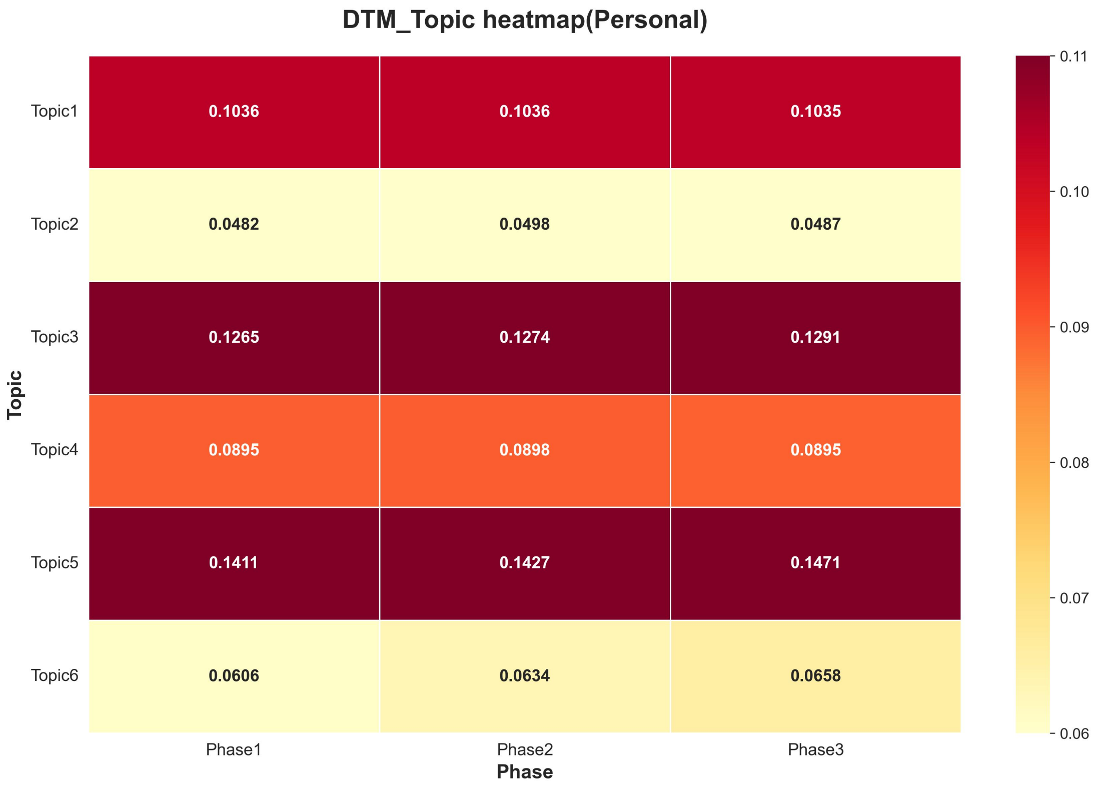

Fig 10. DTM_topic heat map(Personal).

TIFF

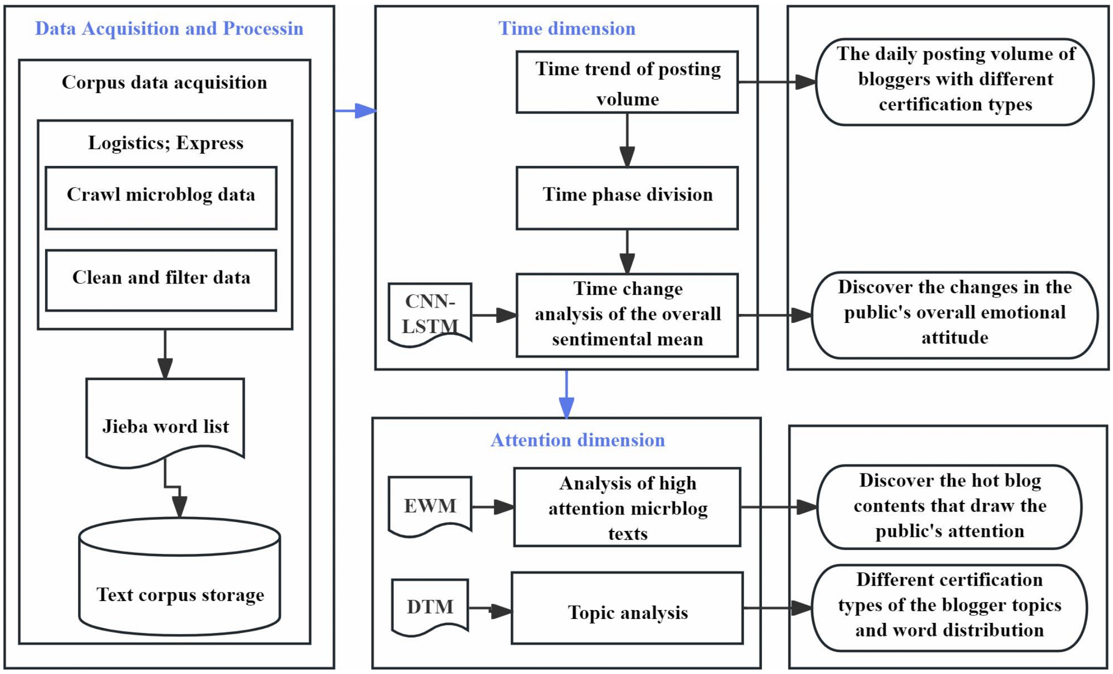

Fig 1. the overall theoretical framework.

TIFF

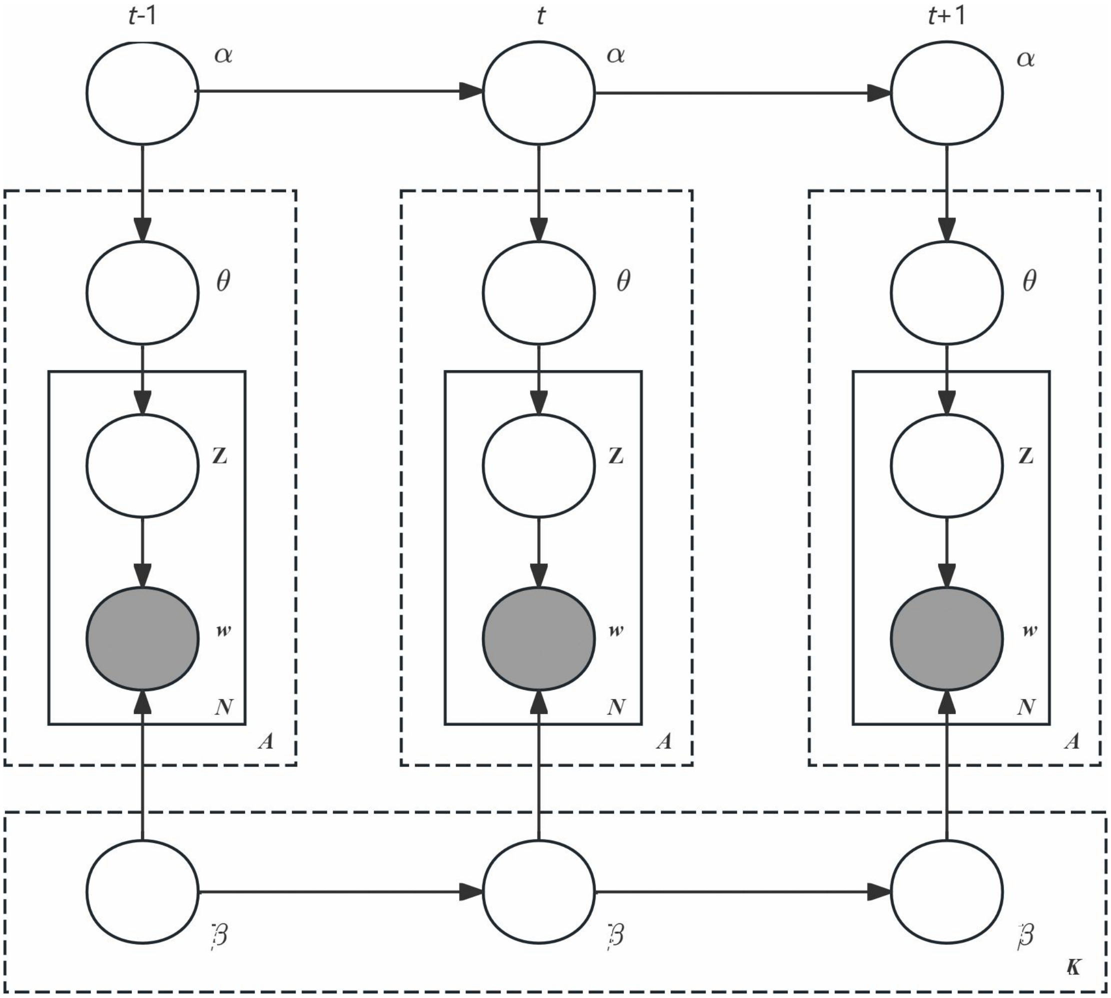

Fig 2. Dynamic topic modeling.

TIFF

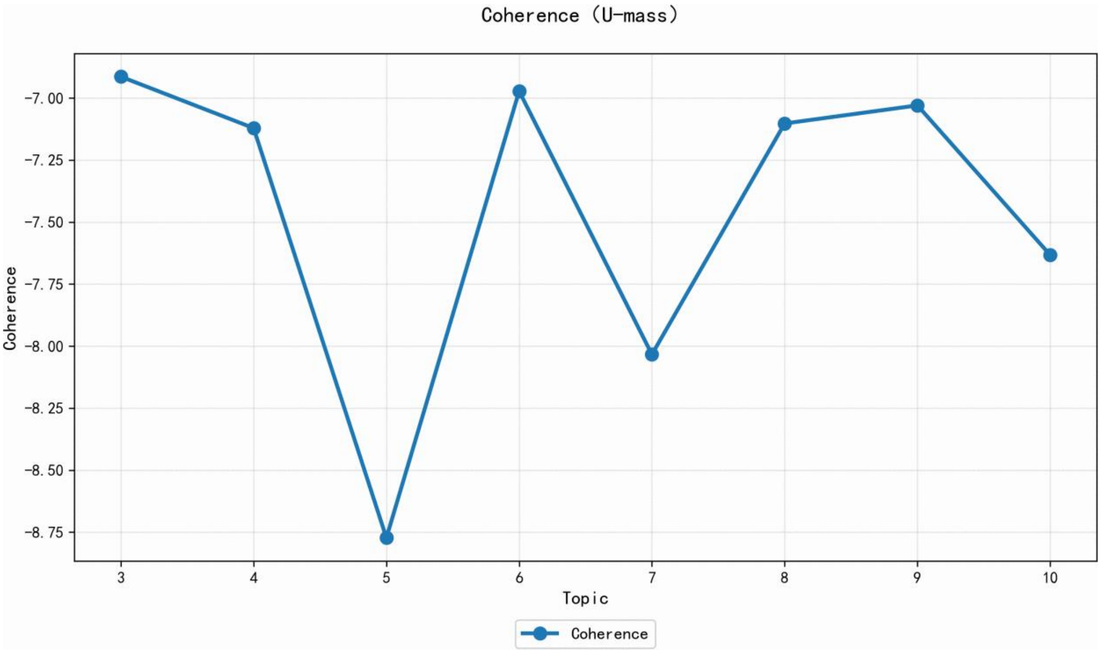

Fig 3. DTM topic-coherence change graph.

TIFF

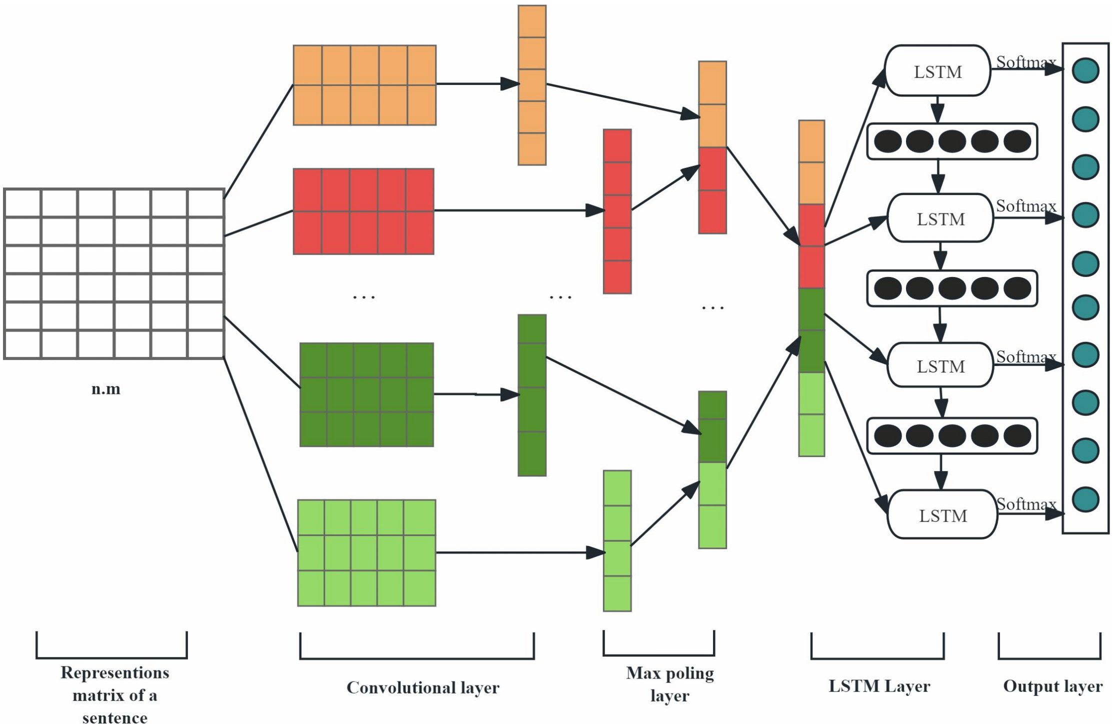

Fig 4. CNN–LSTM model structure diagram.

TIFF

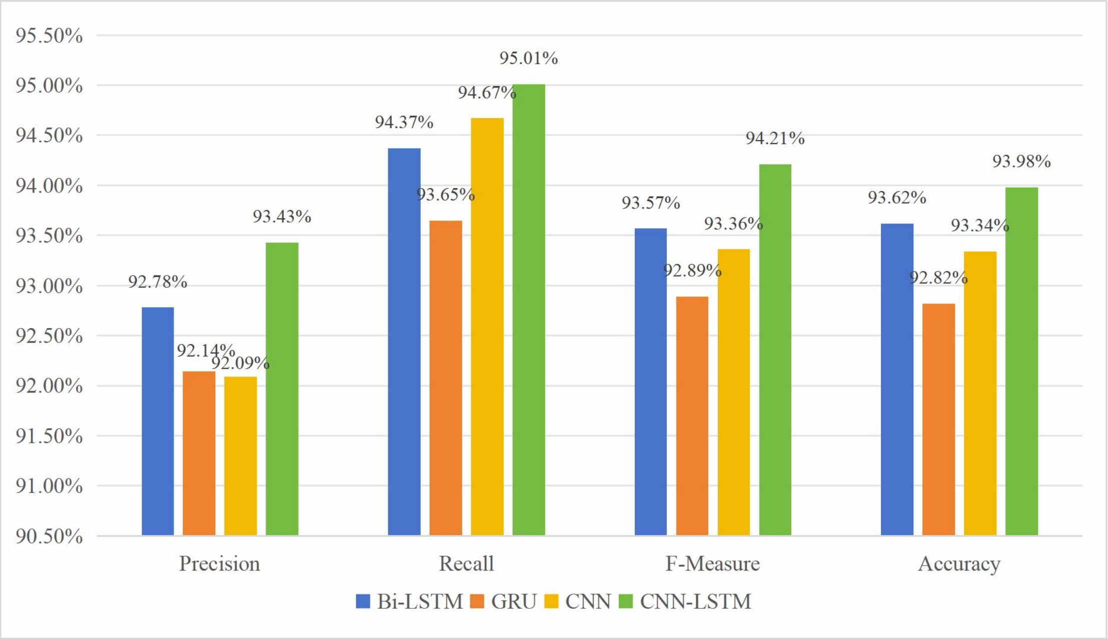

Fig 5. Comparison of model training results. The performance of Bi-LSTM, CRU, CNN, and CNN-LSTM is compared in terms of precision, recall, F-measure and accuracy, with CNN-LSTM achieving the best overall performance.

TIFF

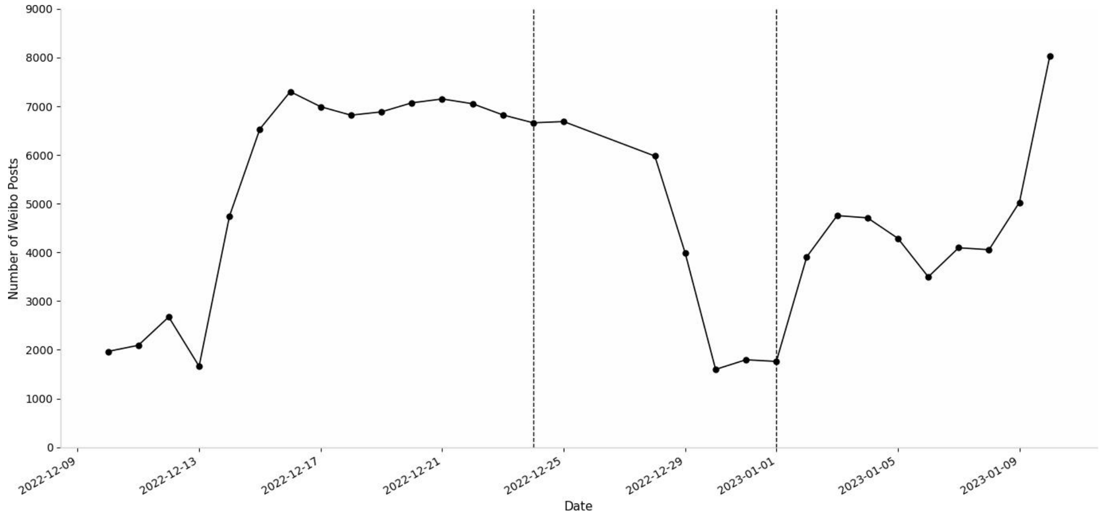

Fig 6. The daily posting volume of the all bloggers. Time series of daily Weibo posting volume from December 2022 to January 2023, showing two major peaks (December 10-23, 2022 and January 2-10, 2023) and a trough from December 24, 2022, to January 1, 2023.

TIFF

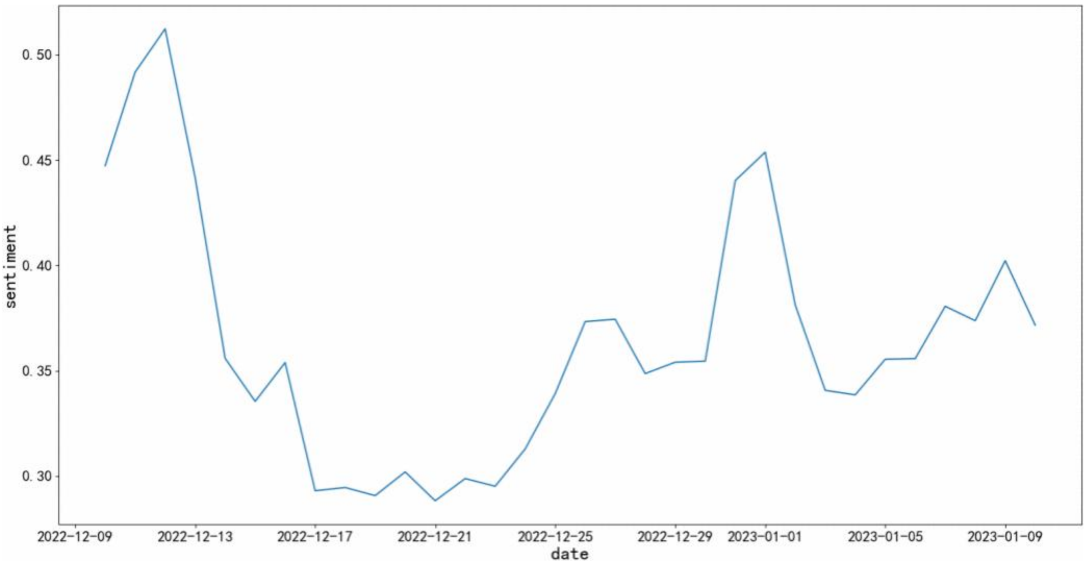

Fig 7. Time-varying graph of the average sentiment value of microblog texts. The sentiment value fluctuated within the range of 0.29-0.51, showing an overall negative trend, with two obvious emotional peaks corresponding to early December and around New Year's Day respectively.

TIFF

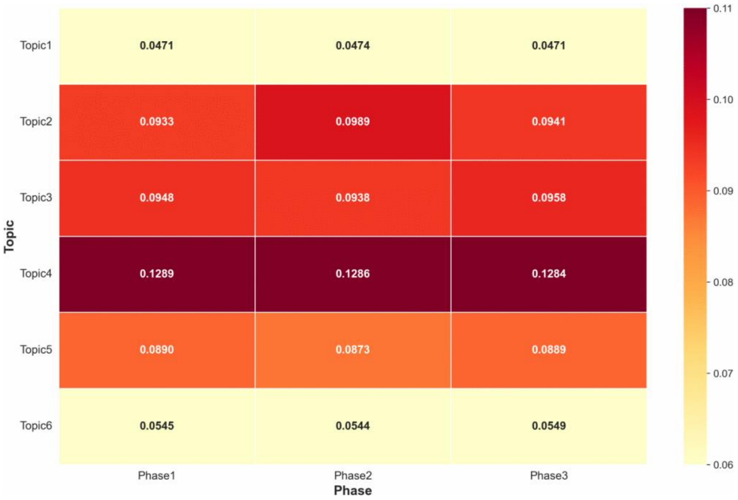

Fig 8. DTM_topic heat map(All).

TIFF

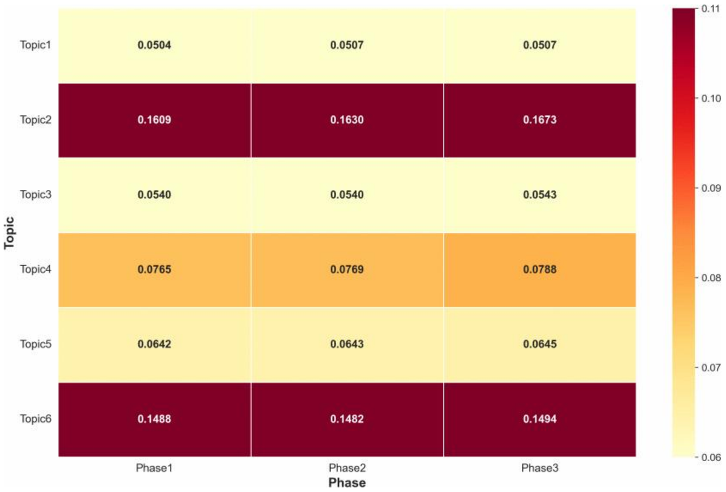

Fig 9. DTM_topic heat map(Official).

TIFF

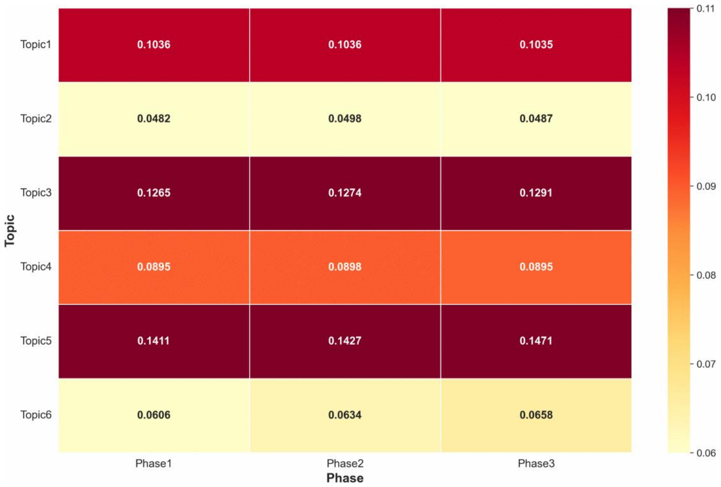

Fig 10. DTM_topic heat map(Personal).
